# Cdyl2-60aa encoded by CircCDYL2 accelerates cardiomyocyte death by blocking APAF1 ubiquitination in rats

**DOI:** 10.1038/s12276-023-00983-5

**Published:** 2023-04-03

**Authors:** Yunfei Deng, Xiaochen Zeng, Yifei Lv, Zhiyuan Qian, Peijie Guo, Yi Liu, Shaoliang Chen

**Affiliations:** 1 Department of Cardiology, Nanjing First Hospital, Nanjing Medical University, Nanjing, China; 2 Department of Clinical Laboratory, Children’s Hospital of Nanjing Medical University, Nanjing, China; 3 Department of Functional Examination, Nanjing First Hospital, Nanjing Medical University, Nanjing, China; 4 Department of Cell Biology, University of California, Davis, CA USA

**Keywords:** Apoptosis, Heart failure

## Abstract

The loss of cardiomyocytes (CMs) after myocardial infarction (MI) is widely acknowledged to initiate the development of heart failure (HF). Herein, we found that circCDYL2 (583 nt) derived from chromodomain Y-like 2 (Cdyl2) is significantly upregulated in vitro (oxygen-glucose deprivation (OGD)-treated CMs) and in vivo (failing heart post-MI) and can be translated into a polypeptide termed Cdyl2-60aa (~7 kDa) in the presence of internal ribosomal entry sites (IRES). Downregulation of circCDYL2 significantly decreased the loss of OGD-treated CMs or the infarcted area of the heart post-MI. Additionally, elevated circCDYL2 significantly accelerated CM apoptosis via Cdyl2-60aa. We then discovered that Cdyl2-60aa could stabilize protein apoptotic protease activating factor-1 (APAF1) and promote CM apoptosis; heat shock protein 70 (HSP70) mediated APAF1 degradation in CMs by ubiquitinating APAF1, which Cdyl2-60aa could competitively block. In conclusion, our work substantiated the claim that circCDYL2 could promote CM apoptosis via Cdyl2-60aa, which enhanced APAF1 stability by blocking its ubiquitination by HSP70, suggesting that it is a therapeutic target for HF post-MI in rats.

## Heart disease: circular RNA linked to heart cell death

The production of a circular RNA molecule called circCDYL2 and the protein it codes for is significantly increased in rat heart muscle cells following loss of blood supply after a heart attack, suggesting new targets for treatment. The circCDYL2 RNA carries the genetic instructions to make a small protein called Cdyl2-60aa. Shaoliang Chen and colleagues at Nanjing Medical University in China detected the increased circCDYL2 in cells from rats with heart failure, and also in cultured rat heart muscle cells experimentally deprived of oxygen and glucose to simulate heart failure. Their research revealed details of the molecular mechanisms allowing Cdyl2-60aa to be produced and then to accelerate the death of heart muscle cells. If applicable to humans, the findings suggest new treatment possibilities by targeting the activity or production of the Cdyl2-60aa protein.

## Introduction

Heart failure (HF), a leading cause of death worldwide, can be categorized into 3 subtypes, namely, HFrEF (heart failure with reduced ejection fraction (EF)), HFmrEF (mid-range EF) and HFpEF (preserved EF), according to the EF values^1^. It is well established that ischemic cardiomyopathy is the main cause of HFrEF and is secondary to myocardial infarction (MI). The subsequent loss of cardiomyocytes (CMs) is the initiation step for HF development. Given the limited regeneration ability of CMs, inhibiting the loss of CMs represents a promising therapeutic strategy for HF.

CircRNA, a newly discovered noncoding RNA, is produced by back-splicing from exons with a covalent bond, is mainly located in the cytoplasm and exerts its function by sponging miRNAs^2^. Current evidence suggests that circRNA can resist exonuclease digestion due to the lack of a 5′ cap and a 3′ poly (A) tail, making it much more stable than linear RNA^3^. There is ample literature suggesting that circRNA is closely related to diverse cardiovascular diseases, such as ischemia/reperfusion injury, cardiac fibrosis or atherosclerosis^4–6^. However, only two studies have hitherto explored the expression profiles of circRNA in failing hearts (one involves HF post-MI, another involves HF post transverse aortic constriction), yielding inconsistent results^7,8^.

The coding potential of circRNA remains subject to debate since it mainly consists of exons. To date, two mechanisms—internal ribosomal entry sites (IRES) and RNA methylation (m6A)—have been demonstrated to initiate circRNA translation^9,10^. More recently, Yibing Yang et al. documented that a polypeptide translated by a circRNA from the FBXW7 gene has potential prognostic implications in brain cancer^11^. Another protein encoded by circSHPRH has been shown to suppress glioma tumorigenesis^12^. Nonetheless, few studies have focused on the relationship between polypeptides encoded by circRNA and the loss of CMs post-MI.

In this work, we discovered that levels of a circRNA (circCDYL2) encoded by the chromodomain Y-like 2 (Cdyl2) gene increased significantly in failing hearts or oxygen-glucose deprivation (OGD)-treated CMs. Moreover, an ~7 kDa polypeptide (Cdyl2-60aa) translated from circCDYL2 accelerated cell death by stabilizing protein Apoptotic protease activating factor 1 (APAF1). Moreover, the reduction in circCDYL2 expression in vivo attenuated the worsening of cardiac function post-MI.

## Materials and methods

### Materials

Anti-Parp-1 (ab74290), anti-cleaved-Parp-1 (ab32064), anti-Caspase 3 (ab13847), anti-cleaved-Caspase 3 (ab2302), anti-β-actin, anti-APAF1 (ab2001), anti-pan-AKT (ab8805), anti-HSP90-beta (ab203085), anti-Ki67 (ab16667), anti-Caspase 9 (ab184786), anti-FKHR (ab179540), anti-IKK-beta (ab124957), anti-p70S6K (ab32529), anti-P65 (ab16502), anti-HSP70 (ab5439), anti-HSC70 (ab51052), anti-IgG (ab133470), anti-HA (ab236632), anti-His (ab18184), Dactinomycin and ADP/ATP Ratio Assay Kits were purchased from Abcam (Britain). TRIzol reagent was acquired from Invitrogen (USA); the SYBR RT‒PCR Kit and DNA PCR kit were from Takara Bio Inc. (Japan); RNase R was from Epicenter (USA).

Primers and siRNAs were designed and synthesized by Sangon Biotech (China). The ATP Assay Kit was purchased from Beyotime (China). The dual-luciferase reporter assay system was obtained from Promega (USA).

### Animal model and samples

A total of 12 male α-MHC-Cre transgenic SD rats (6 weeks old, weighing ~200 g) were obtained from the Animal Core Facility of Nanjing Medical University (Nanjing, China). All animals were housed in a controlled environment with a temperature of 22 ± 1 °C, relative humidity of 50 ± 1%, and a light/dark cycle of 12/12 h. All animal experiments conformed to the Guide for the Care and Use of Laboratory Animals [National Institutes of Health (NIH), Bethesda, MD, USA] and the local ethics review board. Male rats were obtained from the Model Animal Research Center of Nanjing University (Nanjing, China).

All animals were anesthetized with intraperitoneal sodium pentobarbital (50 mg/kg body weight), intubated with a 16-gauge trachea cannula and ventilated with an animal respirator. The left anterior descending coronary artery (LAD) was then ligated with a 6–0 nylon silk suture after the heart was exposed. The MI model was considered successfully established when there was a change in color (pallor was observed on the anterior wall of the left coronary artery). Adeno-associated virus (AAV) is an efficient and safe vector for in vivo gene transfer experiments, and serotype 9 is cardiotropic and has been widely used. Additionally, the Cre-Flex system was utilized to knock down circCDYL2 in hearts. Twelve SD male rats (α-MHC-Cre, 6 weeks old) were randomly categorized into 2 groups (6 rats/group): a negative control (NC) group (surgery and *AAV9-flex-NC)* and an experimental (Ex) group (surgery and *AAV9-flex-sh-circCDYL2)*, which were treated with 5*10^11^ viral genomes (vg) containing sh-circCDYL2 (Obio Technology, Shanghai, China) or empty vector before LAD ligation via the tail vein. After 6 weeks, all rats underwent echocardiographic evaluations with anesthesia via isoflurane inhalation (induction: 4%; maintenance: 2%). The rats were then sacrificed by cervical dislocation. All samples were stored in liquid nitrogen for further use. All efforts were made to minimize animal suffering.

### Circular RNA high-throughput sequencing and computational analysis

RNA was sent to LC-Bio (Hangzhou China) for high-throughput sequencing, performed on an Illumina HiSeq 3000 platform in PE150 sequencing mode. Quantile normalization and subsequent data processing were performed using the R software package and analyzed by LC-Bio. The computational pipeline CIRC explorer was used to obtain back-spliced junction reads for circRNA prediction; the expression abundance of circRNA was measured based on back-spliced reads per million mapped reads (reference genome Rnor_6.0).

### Echocardiography

Echocardiography was performed 6 weeks after MI induction with a 14-Hz ultrasound probe (Hewlett Packard Sonos 5500, USA). The left ventricular 2D image-guided M-mode curves were collected from 6 cardiac cycles for analysis. The heart rate, left ventricular end-systolic diameter (LVESD) and left ventricular end-diastolic dimension (LVEDD) were measured. The left ventricular short-axis ejection fraction (EF) was calculated as follows: EF (%) = [(EDD^3^ − ESD^3^)/EDD^3^] × 100. All parameters were measured over 6 consecutive cardiac cycles and performed by one experienced echocardiographer blinded to the treatment protocol. These data were collected blindly.

### Fluorescence-activated cell sorting (FACS)

To analyze the effects of the indicated treatments on cell survival, we stained the cells with an Annexin V-FITC and PI Detection Kit and analyzed them by FACS. Briefly, trypsinized cells (6 × 10^5^) were gently washed with serum-containing medium followed by PBS. The cells were then resuspended in 100 μl of binding buffer containing 5 μl of annexin V-FITC and 5 μl of PI in the dark for 10 min at room temperature, after which another 400 μl of binding buffer was added to the mixture. The cells were then analyzed by FACS within 1 h after halting the reaction. NRCMs stained with annexin V, PI, or both were designated as early apoptotic, necrotic or late apoptotic cells, respectively. The data were analyzed using BD FACSDiva Software v7.0 (Becton-Dickinson, USA). The total percentage of apoptotic cells was the sum of early and late apoptotic cells. A total of 10,000 cells per group were counted for statistical analysis, and each assessment was repeated three times.

### Cell culture and cell treatments

Primary neonatal rat cardiomyocytes (NRCMs) were isolated as previously described^13^. Briefly, 1- to 3-day-old SD rats were euthanized by decapitation, their hearts were collected, and the ventricles were minced and digested in 0.1% collagenase. Cell suspensions were then collected, centrifuged, resuspended in DMEM with 10% FBS, 100 U/ml penicillin and 100 μg/ml streptomycin, and plated for 1.5 h under standard culture conditions (humidified atmosphere containing 5% CO_2_/95% air at 37 °C), which allows fibroblast attachment to the culture plates. Consequently, cells in suspension (mostly NRCMs) were collected and cultured for an additional 24 h. Monoclonal antibodies against α-actin were used to positively identify cardiac myocytes. Cell purity was confirmed by examining cellular morphology (beating cells) and immunostaining, revealing that the percent of NRCMs was over 90%^13^.

### RNA extraction, RNase R digestion and quality control

Total RNA was extracted from each sample using a homogenizer and TRIzol reagent (Takara Bio Inc., Japan). Subsequently, the quantification and quality of purified RNA were assessed using a NanoDrop ND-1000 (NanoDrop Technologies; Thermo Fisher Scientific, Inc.). The structurally stable circRNA could tolerate the digestion of exonuclease. RNase R is a magnesium-dependent 3´→5′ exoribonuclease that digests all linear RNAs but not lariat or circular RNA structures^14,15^. Accordingly, RNase R (Epicenter, USA; 3 units of RNase R for 1 mg RNA) was applied to total RNA for 30 min at 37 °C to verify the existence of circCDYL2. The integrity of RNA was assessed by electrophoresis on a denaturing 1.5% agarose gel.

### Quantitative real-time polymerase chain reaction (qRT‒PCR) analysis and reverse-transcription PCR (RT‒PCR) analysis

Total RNA isolated from tissues and cells was reverse transcribed into cDNA for qRT‒PCR analysis (Takara Bio Inc., Japan), which was conducted in ABI QuantStudio™ 6 Flex Real-time PCR systems according to the manufacturer’s instructions. A relative expression (the reference gene is β-actin) of 2^(−ΔΔCT)^ compared to the value of the control was used to analyze the gene expression. Agarose gel electrophoresis was used to analyze the results of RT‒PCR. The following primers were used for the experiment: circCDYL2 (F1/R1) 5′-ATCAGCCAGATTTGGAGTTG/CCATTTCCCCTTCTTGTTCT-3′, linear Cdyl2 (F2/R2) 5′- GGCTCTGCTCTGACCAACGG/AAGAATGTGTGTGAAGCCCT-3′, APAF1-5′- TAAGTATGTTATCCCTGTGG/AGTATGCCCAGCGATT-3′, β-actin-5′- CCCATCTATGAGGGTTACGC/TTTAATGTCACGCACGATTTC -3′.

### MiRNAs, plasmids and transfection

Rluc and Fluc reporters with independent start and stop codons were directly connected in the empty vector. Wild-type and mutant IRES sequences of circCDYL2 were obtained through chemical gene synthesis. Rluc-IRES-Fluc frames were obtained via overlap PCR and were then cloned into the *pCMV-HA* vector. The ORF of circCDYL2 plus His tag was cloned into the *pGV486* vector to generate the circRNA. The IRES sequence was mutated in the negative control plasmid, and the *pCMV-Cdyl2-60aa* (*ad- Cdyl2-60aa*) linear overexpression vector was cloned as a positive control. The siRNAs were used to decrease the levels of circCDYL2 and APAF1. Moreover, we utilized 2 different siRNAs targeting one gene to avoid *off-target* effects. *si-APAF1-1*: UGGAUUUGUACCAUUCUUCUG; *si-APAF1-2*: ACUCAUUGGUUCCUUUAAGGG. *si-circCDYL2-1*: UCAACAUUCUCCACCAGUGCA; *si-circCDYL2-2*: CACAAUCCUUUCAACCAAUUC. *sh-circCDYL2*: UCAACAUUCUCCACCAGUGCAUUCAAGAGAUGCACUGGUGGAGAAUGUUGATTTTTT. The siRNAs were transfected with Lipofectamine 3000 (Invitrogen, Carlsbad, CA) according to the manufacturer’s instructions.

### Immunoprecipitation (IP)

Cells were lysed in co-IP buffer (10 mM HEPES [pH 8.0], 300 mM NaCl, 0.1 mM EDTA, 20% glycerol, 0.2% NP-40, protease and phosphatase inhibitors). The lysates were then centrifuged and cleared via incubation with 25 μl protein A/G agarose (MCE, China) for 1.5 h at 4 °C. The precleared supernatant was subjected to IP using the indicated primary antibodies at 4 °C overnight. Then, the protein complexes were collected via incubation with 30 μl protein A/G gel for 2 h at 4 °C. The collected protein complexes were analyzed by immunoblotting.

### Apoptosis-antibody array

NRCMs were treated with *ad-Cdyl2-60aa* or *ad-NC* for 48 h. The cell lysates were then obtained and applied to an Apoptosis-Antibody Array (PAP247; Full Moon Biosystems, CA, USA). The array experiment was performed by Wayen Biotechnologies (Shanghai) Inc. according to the manufacturer’s protocol. The array contained 247 highly specific antibodies related to apoptosis and cell death, each of which had 6 replicates. The slide was scanned on a GenePix 4000B scanner (Axon Instruments, USA). The fluorescence intensity of each array spot was quantified, and the mean value was calculated. The 95% CIs were used to quantify the precision of the phosphorylation ratio based on the analysis of the replicates.

### Northern blotting

Northern blotting was performed according to the manufacturer’s instructions (DIG Northern Starter Kit, Roche). Digoxigenin (Dig)-labeled antisense probes targeting the junction of circCDYL2 were designed by Sangon Biotech (China). In brief, 5 µg of total RNA was resolved on a denaturing urea polyacrylamide gel, transferred to a nylon membrane (Roche) and UV-crosslinked using the standard manufacturer’s protocol. The membrane was then hybridized with specific Dig-labeled RNA probes.

### TTC staining and histological analysis

To analyze the percentage of cardiac infarct area in hearts post-MI (Supplementary Fig. 4l), hearts were excised and sectioned transversely into six sections and then incubated in 2% triphenyltetrazolium chloride (TTC, Sigma‒Aldrich, USA) for 10 min at 37 °C, followed by 10% neutral-buffered formaldehyde for 24 h. Sections were weighed and photographed using a Leica microscope and then analyzed using ImageJ (National Institutes of Health). The viable myocardium was stained red, and the infarcted areas appeared pale. The size of infarction was determined using the following equations: Weight of infarction = (A1 x W1) + (A2 x W2) + (A3 x W3) + (A4 x W4) + (A5 x W5) + (A6 x W6), where A is percent area of infarction by planimetry and W is the weight of each section. Percentage of infarcted left ventricle = (weight of infarction/weight of LV) ×100.

### Immunoblotting

The cells were washed with PBS, lysed on ice in lysis buffer supplemented with protease and phosphatase inhibitors and then centrifuged at 12,000 × *g* for 15 min at 4 °C. The resulting cell lysates were resolved on 7.5% or 12.5% SDS‒PAGE gels and then transferred to PVDF membranes. These membranes were blocked in 5% nonfat dry milk in TBST with 0.1% Tween-20 for 1.5 h at room temperature before the membranes were incubated with the appropriate primary antibodies overnight at 4 °C. The membranes were subsequently washed and incubated with a horseradish peroxidase (HRP)-conjugated secondary antibody for 1 h at room temperature, after which the antibody complexes were visualized and quantified using a chemiluminescence western blotting detection system (Tanon, Shanghai, China). The protein expression levels of the target genes were quantified by relative densitometry and normalized to bands corresponding to β-actin, which was used as an internal control. The results were analyzed by ImageJ. The experiment was run in triplicate.

### RNA fluorescence in situ hybridization (FISH)

A probe targeting the junction of circCDYL2 was used in this assay. Cells were seeded on glass coverslips in 12-well plates. The cells were washed in PBS, fixed in 4% paraformaldehyde (PFA) for 15 min, and then permeabilized in 0.1% Triton X-100 for 15 min. Next, the cells were treated with 30% H_2_O_2_ for 1 h. After washing three times with PBS, cells were further treated with Proteinase K (10 µg/ml) for 25 min. Glycine (2 mg/ml) was used to stop the Proteinase K digestion, followed by refixation with 4% PFA for 20 min. For FISH, cells were incubated at 50 °C in a solution containing 50% formamide, 2× SSC, 0.25 mg/ml Escherichia coli transfer RNA, 0.25 mg/ml salmon sperm DNA (Invitrogen, Carlsbad, CA), 2.5 mg/ml BSA (Roche, Indianapolis, IN), and fluorescently labeled circular probe and linear probes at 125 nM (Sangon Biotech, Shanghai, China). After 12 h, the cells were washed twice for 20 min at 50 °C in 50% formamide and 2× SSC followed by four 5-min washes in PBS (with the penultimate wash containing 4,6-diamidino-2-phenylindole (DAPI)) and an additional brief wash in nuclease-free water.

### Quantification and statistical analysis

All data acquisition and analyses were performed by investigators blinded to the experimental groups. For biochemical analyses, a minimum of four samples per genotype were used for each analysis, while in vivo analysis included at least six rats per genotype. These sample sizes are sufficient to determine whether there is a biologically meaningful difference between different genotypes based on the rat-to-rat variation in energy metabolism and cell apoptosis assessments established in previous studies. For in vitro studies, a sufficiently large number of cells were analyzed to ensure the description of biologically meaningful differences, also following the methods of studies cited throughout the paper. Moreover, the results obtained in cells were reliably reproduced in at least three independent experiments. All experimental data are expressed as the mean ± s.d. unless otherwise mentioned. The normality of the variables was tested by the Shapiro–Wilk test. The data from the analysis met the assumptions of the tests, and the variance was similar between the experimental groups (if not, the data were not included in the following analysis). Unpaired two-tailed Student’s *t* test was used when comparing two experimental groups, while three experimental groups were analyzed using one-way ANOVA followed by Tukey’s post hoc test. Prism version 7.0 (GraphPad Software Inc.) was used for calculations, and *P* values lower than 0.05 were considered significant. Data were recorded and analyzed with SPSS 22.0 (IBM, Chicago, IL, USA), and a *P* value less than 0.05 was considered statistically significant.

## Results

### The identification of circCDYL2 in failing hearts post-MI

**Fig. 1:**
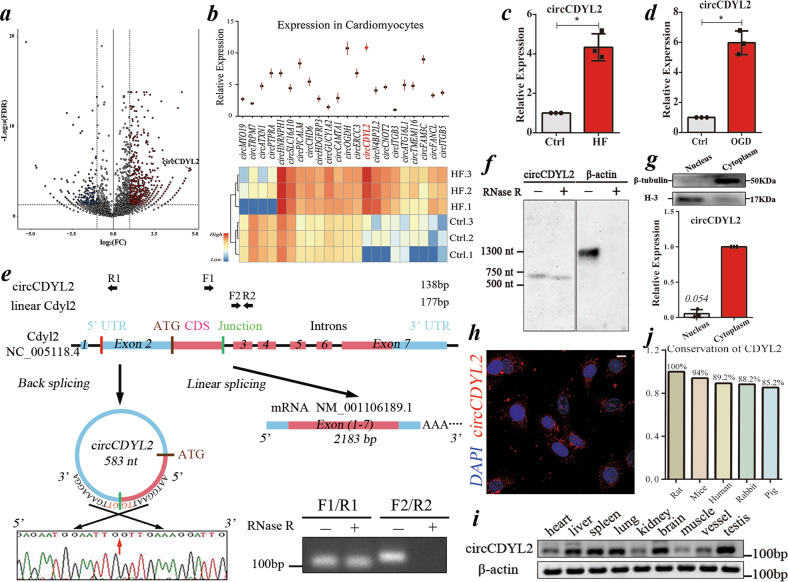
The identification of circCDYL2. **a** Volcano plot showing the differentially expressed circRNAs between the Ctrl and HF groups. The black dot indicates circCDYL2. **b** Upper: qRT-PCT analysis of the expression level of 22 filtered circRNAs in NRCMs; Lower: Heatmap showing the relative expression of 22 circRNAs in 2 groups according to RNA-seq data. Data are expressed as the mean ± s.d., *n* = 3. **c**, **d** qRT-PCR analysis of circCDYL2 in failing hearts (**c**) and OGD-treated NRCMs (**d**). Data are expressed as the mean ± s.d., *n* = 3, *p < 0.01. **e** Schematic of circCDYL2 derived from the Cdyl2 gene (upper) and the results of Sanger sequencing (lower left) and RNase R digestion (lower right). **f** Northern blot analysis of circCDYL2 in NRCMs treated with RNase R using a probe targeting its junction. **g** Upper: Immunoblot of β-tubulin (marker of cytoplasm) and H-3 (marker of nucleus); Lower: qRT‒PCR analysis of the relative expression of circCDYL2 in the nucleus (0.054) and cytoplasm (1). Data are expressed as the mean ± s.d., *n* = 3. **h** FISH assay of circCDYL2 in NRCMs with probes targeting the junction conjugated with PE (phycoerythrin). Scale bar = 10 µm. **i** Semiquantitative PCR (F1/R1) analysis of the distribution of circCDYL2 in diverse organs. **j** Conservation analysis of the CDYL2 protein in different species.

Ligation of the left anterior descending (LAD) coronary artery was used to establish rat HF models evidenced by echocardiography. The control (Ctrl) and HF groups (3 rats per group) were set up (Supplementary Fig. 1a). The hearts were harvested at 4 weeks post ligation and underwent high-throughput sequencing by LC-Bio (Hangzhou, China) to identify circRNAs. Overall, 3682, 3346, and 3425 circRNAs in the Ctrl group and 3820, 3592, and 3328 circRNAs in the HF group were found, respectively. A total of 795 differentially expressed (aFC (fold change) >2.0 and *p* < 0.05) circRNAs were identified (611 upregulated and 184 downregulated circRNAs in the HF groups) (Fig. 1a).

Considering that the circRNA expression levels partly determined their role in the heart, 795 circRNAs were further screened with a stringent criterion: reads per million mapped reads (RPM) >1 and number of mapped junctions (NMJ) >20. Finally, 22 circRNAs were screened, and a circRNA (circCDYL2, derived from the second exon of the Cdyl2 gene; 583 nt) attracted our attention for the following reasons: (1) It had a higher expression level compared to the other 21 candidates in CMs (over 0.1% relative to β-actin and ~3% relative to linear Cdyl2 (mRNA)) (Fig. 1a, b; Supplementary Fig. 1b); (2) It was significantly increased in vivo (failing hearts) and in vitro (oxygen-glucose deprivation (94% N_2_, 5% CO_2_ and 1% O_2_ + glucose <1 g/L)-treated neonatal rat cardiomyocytes (NRCMs)) (Fig. 1c, d). In contrast, no significant change in linear Cdyl2 (F2/R2 in Fig. 1e) was detected in failing hearts or OGD-treated NRCMs, indicating that changes in circCDYL2 were not paralleled by changes in linear Cdyl2 (Supplementary Fig. 1c). Additionally, the circCDYL2 expression level was much lower in fibroblasts than in NRCMs. In contrast, linear Cdyl2 expression in NRCMs was comparable to that in fibroblasts, further supporting the above findings and emphasizing the role of circCDYL2 in CMs (Supplementary Fig. 1d).

The existence of circCDYL2 in CMs was confirmed by Sanger sequencing (RT‒PCR products (F1/R1) containing the junction), RNase R toleration and Northern blotting (targeting the junction) (Fig. 1e, f). The results of nuclear separation and FISH indicated that it was predominantly localized in the cytoplasm (Fig. 1g, h). Moreover, we found that it exists in diverse organs, and the CDYL2 protein is highly conserved across different species (Fig. 1i, j). Altogether, the above results aroused our interest in the role of circCDYL2 in CMs.

### circCDYL2 accelerates the apoptosis of CMs in vitro and in vivo

**Fig. 2:**
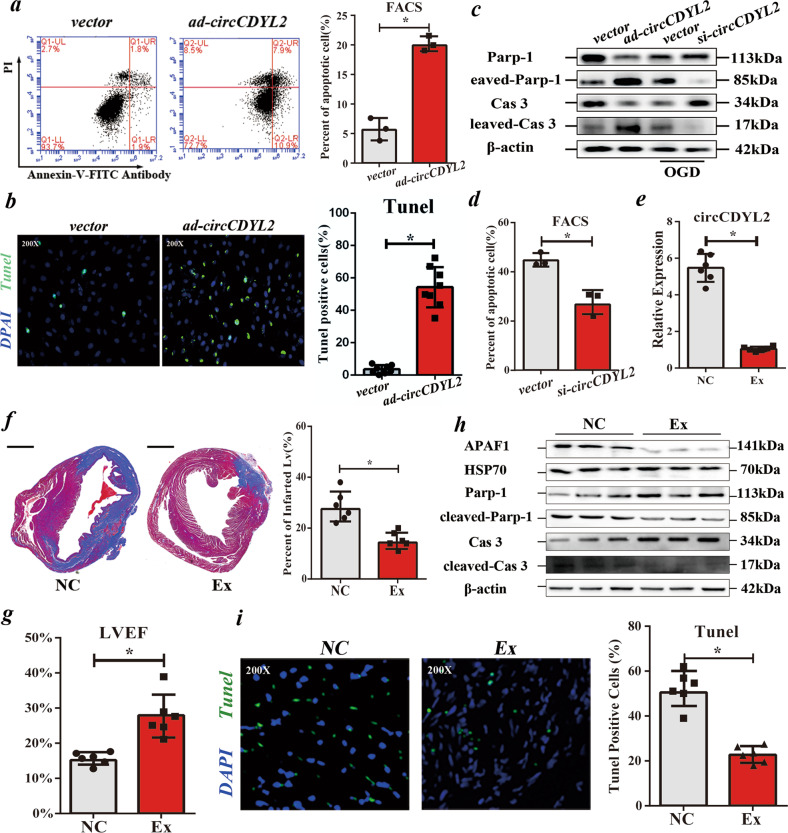
circCDYL2 accelerates the apoptosis of CMs in vitro and in vivo. **a** Left: Representative results of FACS assays of apoptotic NRCMs overexpressing circCDYL2; Right: Quantitative analysis of the results of FACS assays. **b** Left: Representative images (200×) of TUNEL assays showing apoptotic NRCMs overexpressing circCDYL2. Right: Quantitative analysis of the results of TUNEL assays. Data are expressed as the mean ± s.d., *n* = 8, two-tailed *t* test, *p < 0.05. **c** Immunoblot analysis of apoptosis-related proteins (Parp-1 and Caspase 3 (Cas 3)) in NRCMs with over- or low-expression of circCDYL2. **d** FACS analysis of OGD-treated NRCMs with low circCDYL2 expression. **e** qRT‒PCR analysis of circCDYL2 in hearts from NC or Ex (transfected with *AAV9-shRNA* (targeting circCDYL2)) groups. **f** Left: Representative images of heart sections by Trichrome staining (viable myocardium stained in red, and the infarcted areas appeared in blue); Right: Bar graph showing the infarcted LV in NC or Ex groups. Scale bar = 2 mm. **g** Results of LVEF of hearts from 2 groups at 6 weeks after surgery. **h** Immunoblot of APAF1, HSP70, Parp-1 and Caspase 3 in hearts from 2 groups. **i** Left: Representative images (200×) of TUNEL assays showing apoptotic CMs in the two groups. Right: Quantitative analysis of the results of TUNEL assays. **a**, **d**–**i** All data are expressed as the mean ± s.d., *n* = 3 or 6, two-tailed *t* test, *p < 0.05.

To address its role in CMs, we overexpressed and silenced circCDYL2 with the adenovirus vector (to improve the transfection efficiency in the primary cell, *ad-circCDYL2*) and *si-circCDYL2* (targeting the junction), respectively, as described in our previous study^16^. Two *si-circCDYL2s* (targeting different sites) were designed to avoid *off-target* effects. qRT‒PCR indicated that *si-circCDYL2* could effectively knock down circCDYL2 but not linear Cdyl2 (Supplementary Fig. 1e). Moreover, *ad-circCDYL2* significantly upregulated circCDYL2 in NRCMs (Supplementary Fig. 1f). The following assays indicated that circCDYL2 overexpression could accelerate cell apoptosis but had no effects on cell autophagy, pyroptosis, energy metabolism, cell proliferation or cell migration (Fig. 2a–c; Supplementary Fig. 1g–k). circCDYL2 reduction significantly inhibited OGD-treated NRCM apoptosis, proving that circCDYL2 is mainly associated with cell apoptosis (Fig. 2c, d).

To confirm its effects on CMs in vivo, we injected α-MHC-Cre transgenic rats with *AAV9-flex-sh-circCDYL2* (adeno-associated virus vector) or *AAV9-flex-NC* before ligation. qRT‒PCR showed that circCDYL2 was significantly reduced in the experimental group (Ex, injected with *AAV9-flex-sh-circCDYL2*) compared to the negative control group (NC, injected with *AAV9-flex-NC*) (Fig. 2e). A decreased heart infarct area and higher LVEF in the Ex group substantiated better cardiac function in the Ex group (Fig. 2f, g). The reduction in apoptosis-related proteins (cleaved-Parp-1 and cleaved-Caspase 3) and apoptotic CMs in the Ex group indicated that reduced circCDYL2 could inhibit CM apoptosis in vivo (Fig. 2h, i). No obvious effects of circCDYL2 on cell autophagy, pyroptosis, energy metabolism or proliferation were detected, consistent with the in vitro results (Supplementary Fig. 1l, m). In conclusion, circCDYL2 played a vital role in CM apoptosis in vitro and in vivo.

### circCDYL2 possesses translation potential

**Fig. 3:**
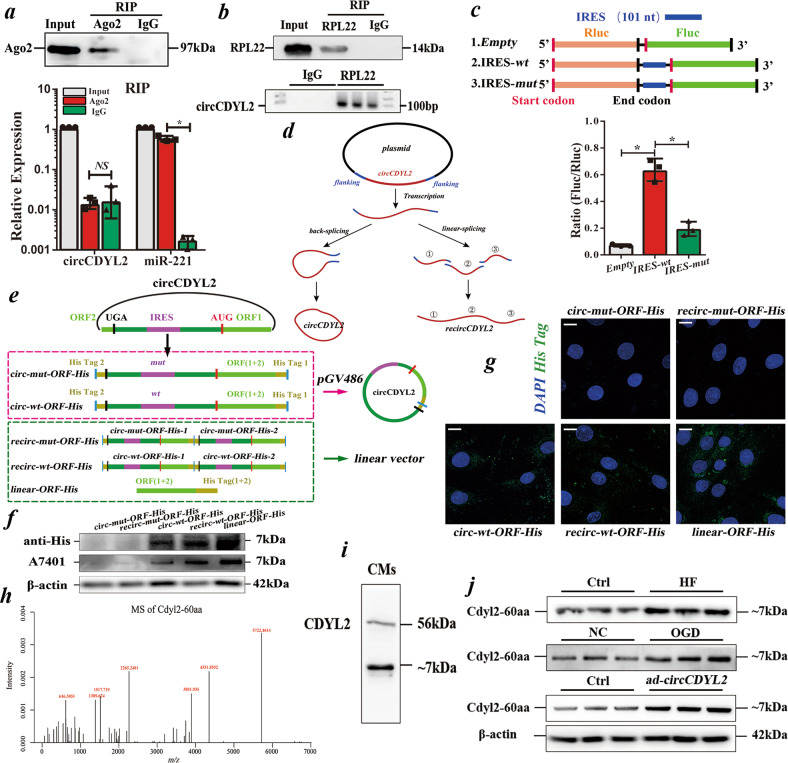
circCDYL2 encodes an ~7 kDa polypeptide. **a** Upper: Immunoblot of Ago2 from RIP (Ago2) assay in NRCMs; Lower: qRT-PCR of circCDYL2 or miR-221 (positive control) from RIP of Ago2. Data are expressed as the mean ± s.d., *n* = 3, two-tailed *t* test, *p < 0.001, NS not significant. **b** Upper: Immunoblot of RPL22 from RIP (RPL22) in NRCMs; Lower: Semiquantitative PCR analysis of circCDYL2 from RIP of RPL22. **c** Upper: Schematic of the vector set in a dual luciferase system with or without the IRES (*wt or mu*t). Lower: The ratio of Fluc/Rluc in NRCMs transfected with *Empty, IRES-wt* or *IRES-mut*. Data are expressed as the mean ± s.d., *n* = 3, two-tailed *t* test, *p < 0.05. **d** Schematic of the mechanism of generating recircCDYL2 (repeated circCDYL2). **e** Schematic of the different sets of *circ-mut-ORF-His, circ-wt-ORF-His, recirc-mut-ORF-His, recirc-wt-ORF-His or linear-ORF-His*. **f** Immunoblot of His tag and antibody A7401 in NRCMs with the above 5 different plasmids. **g** Immunofluorescence of His tag in NRCMs with the above 5 different plasmids. Scale bar = 10 µm. **h** Mass spectrum (MS) analysis of the polypeptide pulled down by anti-His in NRCMs transfected with *circ-wt-ORF-His*. **i** Immunoblot of a commercial CDYL2 antibody recognizing the antigen containing this ~7 kDa polypeptide in NRCMs. **j** Immunoblot analysis of endogenized Cdyl2-60aa in failing hearts, OGD-treated NRCMs or NRCMs with *ad-circCDYL2*.

To explore the mechanism underlying the effect of circCDYL2 on CM apoptosis, we first conducted RNA binding protein immunoprecipitation (RIP) of Ago2 to clarify whether circCDYL2 binds to miRNAs on the premise that circRNA mainly functions as a miRNA sponge. However, qRT‒PCR showed that circCDYL2 could not be pulled down by Ago2 protein, suggesting that it works in CMs in an uncanonical way (Fig. 3a).

To examine whether circCDYL2 binds to proteins, we synthesized biotin-labeled circCDYL2 to pull down the associated proteins. Mass spectrometry (MS) analysis indicated that the pulled-down proteins (RPL22, RPSA, and so on) were mainly associated with the ribosome-nascent chain complex (RNC) (Supplementary Table 1). RIP of RPL22 confirmed that circCDYL2 could bind to RNCs, suggesting that it might participate in the translation process (Fig. 3b). It is widely acknowledged that two mechanisms, IRES (internal ribosomal entry sites) and circRNA methylation (m6A, RRACH (R = G or A; H = A, C or U)), can initiate circRNA translation^9,10^. We then found that circCDYL2 contains one highly conserved open reading frame (ORF, https://www.ncbi.nlm.nih.gov/orffinder/) of 183 nt encoding a 60 aa polypeptide and an IRES (*RNAfold* (http://rna.tbi.univie.ac.at/cgi-bin/RNAWebSuite/RNAfold.cgi)), but no m6A domain between the start and end codons, further supporting the above hypothesis (Supplementary Fig. 2/3a).

### circCDYL2 encodes an ~7 kDa polypeptide

To address whether IRES initiates the translation of circCDYL2, we first assessed its activity. We cloned the wild-type (*wt*) or mutant (*mut*) IRES (101 nt) between the Rluc and Fluc reporter genes in a dual-luciferase vector system. Rluc and Fluc reporters were directly connected in the empty vector. The intensity ratio of Fluc/Rluc in the *wt* group was much higher than that in the *mut* or *empty* groups, showing that IRES could induce translation in a cap-independent manner (Fig. 3c).

Subsequently, to verify its activity in circCDYL2, we cloned circCDYL2 with *wt* or *mut* IRES into *pGV486*. To add a His-tag, we moved the junction to the stop codon of the ORF and separated the His-tag sequence to both sides (*circ-wt-ORF-His* or *circ-mut-ORF-His*). To avoid the noise signal from circRNA repeats (the byproducts of plasmids via impaired back-splicing (linear-splicing, Fig. 3d)^17^), we constructed *recirc-wt-ORF-His* or *recirc-mut-ORF-His* as the control vector. For the positive control, the linearized ORF plus the His-tag was cloned into a linear vector (*linear-ORF-His*) (Fig. 3e). Immunoblot results showed that the anti-His tag could detect an ~7 kDa polypeptide (Cdyl2-60aa) in CMs transfected with *circ-wt-ORF-His, recirc-wt-ORF-His* and *linear-ORF-His*, which was also confirmed by immunofluorescence of the anti-His tag with confocal imaging and MS (Fig. 3f–h). However, because of the byproducts of *recirc-wt-ORF-His*, it remains unclear whether IRES in circCDYL2 can initiate translation. Importantly, a commercial antibody (A7401 from ABclonal (China), whose antigen contains the ~7 kDa polypeptide) yielded 2 bands in samples from CMs (Fig. 3i). The molecular weight of one band was ~7 kDa, the sequence of which was the same as Cdyl2-60aa according to the MS results (Fig. 3h). Immunoblotting showed that the endogenized ~7 kDa polypeptide increased significantly in failing hearts and OGD-treated NRCMs, consistent with circCDYL2 (Fig. 3j). Additionally, Cdyl2-60aa increased obviously in *ad-circCDYL2-*transfected NRCMs (Fig. 3j). The above results showed that circCDYL2 encodes an ~7 kDa polypeptide (Cdyl2-60aa) in vivo and in vitro.

### circCDYL2 affects the apoptosis of CMs via Cdyl2-60aa

**Fig. 4:**
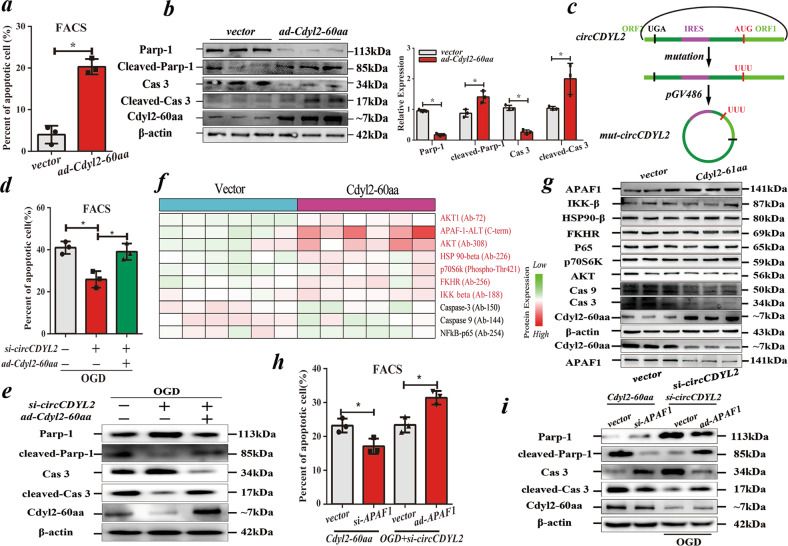
circCDYL2 affects the apoptosis of CMs via the Cdyl2-60aa - APAF1 axis. **a** FACS analysis of apoptotic NRCMs overexpressing Cdyl2-60aa. **b** Immunoblot (left) and quantification (right) analysis of apoptosis-related proteins (Parp-1 and Caspase 3 (Cas 3)) in NRCMs overexpressing Cdyl2-60aa. **c** Schematic of the generation of *mut-circCDYL2* (AUG→UUU). **d** FACS analysis of apoptotic NRCMs cotransfected with *si-circCDYL2* and *ad-Cdyl2-60aa*. **e** Immunoblot of Parp-1 and Caspase 3 (Cas 3) in NRCMs cotransfected with *si-circCDYL2* and *ad-Cdyl2-60aa*. **f** Heatmap showing the results of the apoptosis antibody array of NRCMs overexpressing Cdyl2-60aa. Red refers to 7 upregulated proteins, and black refers to 3 downregulated proteins. **g** Immunoblot of the above 10 filtered proteins in NRCMs overexpressing Cdyl2-60aa. **h** FACS analysis of apoptotic NRCMs cotransfected with *ad-Cdyl2-60aa* and *si-APAF1* or *si-circCDYL2* and *ad-APAF1*. **i** Immunoblot of Parp-1 and Caspase 3 (Cas 3) in NRCMs cotransfected with *ad-Cdyl2-60aa* and *si-APAF1* or *si-circCDYL2* and *ad-APAF1*. **a**, **b**, **d**, **h** All data are expressed as the mean ± s.d., *n* = 3 or 6, two-tailed *t* test, *p < 0.05.

To clarify the role of Cdyl2-60aa in CMs, we utilized *ad-Cdyl2-60aa* to directly upregulate its expression to abrogate the effects of circCDYL2 on CMs. The results showed that it could significantly accelerate apoptosis but had no effects on autophagy, pyroptosis, proliferation or ATP synthesis, similar to circCDYL2 (Fig. 4a, b; Supplementary Fig. 4a–e). Then, we upregulated *mut*-circCDYL2 in NRCMs with the aforementioned *circ-mut-ORF-His* to abrogate the effects of Cdyl2-60aa. Notably, the increased *mut-*circCDYL2 yielded no obvious effects on cell apoptosis (Supplementary Fig. 4f, g). To assess whether the *mut*-IRES in *circ-mut-ORF-His* silences the influence of circCDYL2 on cell apoptosis, we reconstructed *ad-mut-circCDYL2* with an altered starting codon (AUG to UUU) to stop the generation of Cdyl2-60aa (Fig. 4c). Similar phenotypes were detected in NRCMs transfected with *ad-mut-circCDYL2* (data not shown). Finally, we cotransfected NRCMs with *si-circCDYL2* and *ad-Cdyl2-60aa*. The following assays indicated that the decrease in apoptosis caused by *si-circCDYL2* was attenuated by Cdyl2-60aa overexpression, proving that circCDYL2 mainly affects cell apoptosis via Cdyl2-60aa (Fig. 4d, e).

### APAF1 bridges Cdyl2-60aa with cell apoptosis

To investigate how Cdyl2-60aa mediates CM apoptosis, we screened the potential downstream targets utilizing an apoptosis antibody array; the results showed that 10 proteins were significantly altered in Cdyl2-60aa-overexpressing NRCMs (Supplementary Table 2, Fig. 4f)^18^. Next, the immunoblot assay showed that expression of APAF1 was increased and that of Caspase 3/9 was decreased in Cdyl2-60aa-overexpressing NRCMs, consistent with the array results (Fig. 4g). APAF1, a mammalian homolog of cell death protein 4 (CED-4), leads to cell apoptosis by activating caspase 9^19^. Considering that Caspase 3/9 are the downstream targets of APAF1, we hypothesized that APAF1 bridges Cdyl2-60aa with cell apoptosis. We found that the reduction in circCDYL2 inhibited APAF1 expression in vitro and in vivo (Fig. 2h/4g). Then, we cotransfected NRCMs with *si-circCDYL2* and *ad-APAF1* or *ad-Cdyl2-60aa* and *si-APAF1*. The results showed that the protective effects of *si-circCDYL2* on CMs were abrogated by APAF1 overexpression, while the apoptotic effects caused by *ad-Cdyl2-60aa* were rescued by *si-APAF1*, confirming that APAF1 mediates the effects of Cdyl2-60aa on cell apoptosis (Fig. 4h, i).

### Cdyl2-60aa stabilizes APAF1 by blocking its ubiquitination

**Fig. 5:**
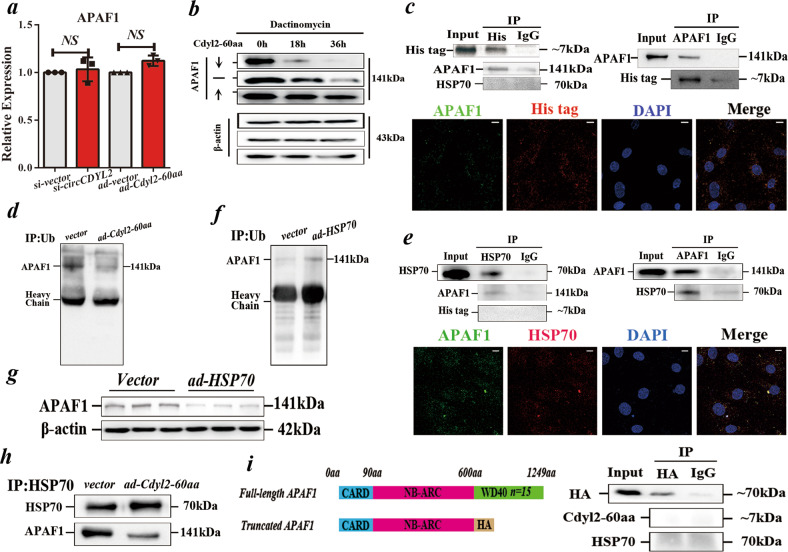
Cdyl2-60aa stabilizes APAF1 by blocking its ubiquitination. **a** qRT-PCR of APAF1 in NRCMs with over- or low-expressed Cdyl2-60aa. Data are expressed as the mean ± s.d., *n* = 3, two-tailed *t* test, NS not significant. **b** Immunoblot of APAF1 in dactinomycin-treated NRCMs with increased or reduced Cdyl2-60aa at different time points (0 h, 18 h and 36 h). **c** IP of His tag (Upper Right) with APAF1 and HSP70; or APAF1 (Upper Left) with His tag in NRCMs transfected with *ad-Cdyl2-60aa* (containing His tag*)*; Immunofluorescence analysis (Lower) of the localization of APAF1 and Cdyl2-60aa in NRCMs. Scale bar = 10 µm. **d** IP of Ub (ubiquitination) with APAF1 in NRCMs transfected with *ad-Cdyl2-60aa*. **e** IP of HSP70 (upper left) with APAF1 and His tag or APAF1 (upper right) with HSP70 in NRCMs transfected with *ad-Cdyl2-60aa* (containing His tag*)*; immunofluorescence analysis (lower) of the localization of APAF1 and HSP70 in NRCMs. Scale bar = 10 µm. **f** IP of Ub with APAF1 in NRCMs overexpressing HSP70. **g** Immunoblot of APAF1 in NRCMs overexpressing HSP70. **h** IP of HSP70 with APAF1 in NRCMs overexpressing Cdyl2-60aa. **i** Left: Schematic of full-length or truncated APAF1. The CARD domain is related to APAF1-mediated cell death. NB-ARC domain is related to RNA binding; Right: IP of HA tag (truncated APAF1, ~70 kDa) with Cdyl2-60aa and HSP70.

Little is known about how Cdyl2-60aa affects APAF1. We first assessed changes in APAF1 at the genetic level in NRCMs with low or overexpressed Cdyl2-60aa, but no variations were detected (Fig. 5a). We then assessed the stability of protein APAF1 in Cdyl2-60aa up- or downregulated NRCMs treated with dactinomycin. The immunoblot assay showed that APAF1 was degraded more slowly in Cdyl2-60aa-overexpressing NRCMs than in the control group, suggesting that Cdyl2-60aa could increase APAF1 expression at the protein level (Fig. 5b). Interestingly, co-IP and immunofluorescence results indicated that Cdyl2-60aa was directly associated with APAF1 (Fig. 5c).

Then, we screened the potential mechanisms related to APAF1 stability. No DEVD motif (the target peptide of caspases) but several KFERQ-like motifs (the signal for lysosomal pathways) were found in protein APAF1 (Supplementary Table 3)^20^. IP of HSC70 (recognizer of KFERQ-like motif) showed that Cdyl2-60aa overexpression did not affect HSC-associated APAF1, suggesting that Cdyl2-60aa might be related to APAF1 ubiquitination (Supplementary Fig. 4h). The finding that less APAF1 is ubiquitinated in Cdyl2-60aa-overexpressing NRCMs confirms that Cdyl2-60aa could affect APAF1 ubiquitination (Fig. 5d). Above all, Cdyl2-60aa was anti-related to APAF1 ubiquitination, indicating that Cdyl2-60aa affects APAF1 ubiquitination indirectly.

Previous research indicated that heat-shock protein 70 (HSP70) could accelerate APAF1 degradation in retinal ischemia injury^20^. HSP70 plays a vital role in the proteome quality process, forming a complex with HSP40 and NEF (nucleotide exchange factor)^21^. It also accelerates protein degradation by acting as a hinge of STUB1 (an E3 ubiquitin transferase) with the target protein. We next verified that APAF1 binds to HSP70 in CMs via IP of APAF1 and HSP70 with pulldown of either protein. The colocalization of HSP70 and APAF1 in NRCMs confirmed their direct binding (Fig. 5e). In addition, an increase in HSP70 could increase the ubiquitination of APAF1 and decrease the level of APAF1. HSP70 levels did not change significantly between the NC and Ex groups, suggesting that HSP70 is associated with APAF1 ubiquitination and degradation in CMs (Fig. 2h/5f, g).

The above results corroborated the idea that Cdyl2-60aa and HSP70 have anti-effects on APAF1 ubiquitination, making us wonder whether they compete for the interaction with APAF1, based on the fact that Cdyl2-60aa and HSP70 cannot pull each other down (Fig. 5c–e). We also discovered that HSP70 pulled down less APAF1 in Cdyl2-60aa-overexpressing CMs (Fig. 5h). By analyzing the structure of APAF1 via *SMART* (http://smart.embl-heidelberg.de/), we found that it consists of several WD40 domains, which mediate diverse protein‒protein interactions (Supplementary Fig. 4i)^22^. After we constructed a WD40-truncated APAF1, neither Cdyl2-60aa nor HSP70 connected to the WD40-truncated APAF1, suggesting that Cdyl2-60aa and HSP70 bind to the same domain of APAF1 (Fig. 5i). In conclusion, the above results demonstrated that Cdyl2-60aa increases the stability of APAF1 by blocking HSP70-mediated ubiquitination.

## Discussion

Cdyl2 is one of the homologs of the CDY gene expressed abundantly in the testis, while Cdyl2 is ubiquitously expressed. The CDY family proteins are mainly located in the nucleus and contain two domains: the chromodomain and the enoyl-coenzyme A hydratase-isomerase catalytic domain (catalytic domain)^23,24^. Overwhelming evidence substantiates the idea that the chromodomain domain is related to the maturation of spermatids by acetylating histone H4 and H2A, while the catalytic domain can bind CoA and histone deacetylases and acts as a corepressor of transcription in somatic cells^25–29^. In our work, we identified circCDYL2 from the second exon of the Cdyl2 gene that was significantly upregulated in failing hearts. Furthermore, we found that circCDYL2 could be translated into a polypeptide (Cdyl2-60aa) that contains the chromodomain domain of CDYL2. However, immunoblotting and immunostaining showed that Cdyl2-60aa is mainly located in the cytoplasm, suggesting that it does not possess the ability of histone acetylation in CMs. Our findings highlight that Cdyl2-60aa plays a vital role in CM apoptosis post-MI by blocking APAF1 ubiquitination.

To upregulate the expression of circCDYL2, we constructed a plasmid that generates circCDYL2 via back-splicing. However, according to the mechanism in Fig. 3d, the repeat circCDYL2 (*recircCDYL2*) sharing the same sequence and junction with circCDYL2 would be generated unavoidably. Here, we transfected *ad-circCDYL2* into HEK293 cells (not containing circCDYL2). Then, gel analysis and Sanger sequencing of the PCR product (F1/R1) from RNase R-treated HEK293 total RNAs showed that some of the *ad-circCDYL2* products were digested, confirming that both circCDYL2 and recircCDYL2 can be generated (Supplementary Fig. 4j). The issue was whether we can distinguish the effects of circCDYL2 from recircCDYL2 on CMs. Importantly, we found that both could generate Cdyl2-60aa via IRES, showing that circCDYL2 and recircCDYL2 exert similar functions in CMs. Consistently, our previous study demonstrated that circSNRK and precircSNRK (precursor circSNRK) played similar roles in CMs acting as miRNA sponges^16^, indicating that circRNA and its concatemer play identical biological roles. Except for their different degradation rates, they were similar.

No obvious change was found in Cdyl2 at either the genetic or protein level in failing hearts or in OGD-treated NRCMs, suggesting that the overexpression of circCDYL2 is mainly associated with increased cyclization of pre-Cdyl2 (pre-mRNA, circCDYL2 and linear Cdyl2 derived from the same pre-Cdyl2). Increased circCDYL2 did not affect the expression of Cdyl2 significantly since circCDYL2 represents only ~3% of linear Cdyl2. Moreover, our previous study showed that the formation of circCDYL2 could be regulated by NOVA alternative splicing regulator 1 (NOVA1)^16^. In the present study, we found that NOVA1 was significantly increased in OGD-treated NRCMs, suggesting that elevated NOVA1 could account for the increase in circCDYL2 to a certain extent (Supplementary Fig. 4k).

CircRNA is a noncoding RNA that lacks coding ability given the absence of 5′ and 3′ terminals. The identification of an IRES in circCDYL2 and subsequent assays confirmed that the IRES could initiate the translation of circCDYL2. Previous studies have documented that truncated polypeptides from circRNA exert a vital role in the pathological process^11,12^. Although the level of circCDYL2 is much lower than that of its linear counterpart, its unique trait of being more stable than linear RNAs amplifies its effects on CMs. Additionally, we found that circCDYL2 can be detected in mice (MMU_CIRCpedia_4128; 583 nt) and humans (HSA_CIRCpedia_18832; 592 nt) according to the CIRCpedia database^30^. To explore their coding potential, we compared their IRES sequences, ORFs and possible translated polypeptides to circCDYL2. The results showed that both of them have a relatively high conservation (Supplementary Fig. 3a–c). However, the secondary structures of IRESs predicted by *RNAfold* indicated that both IRESs might not possess coding ability (Supplementary Fig. 3d). The above hypothesis was confirmed by immunoblot results, highlighting that Cdyl2-60aa being translated by circCDYL2 in a cap-independent manner is an occasional event (Supplementary Fig. 3e). HSP70 and Cdyl2-60aa cannot combine with APAF1 simultaneously, indicating that they compete for the same binding site on APAF1. Subsequent assays showed that both of them can bind to the WD40 domain of APAF1, further supporting the above findings. Cdyl2-60aa disrupted the binding of HSP70 to APAF1 and, in turn, blocked APAF1 ubiquitination, providing a reasonable explanation for Cdyl2-60aa elevating APAF1 expression at the protein level.

**Fig. 6:**
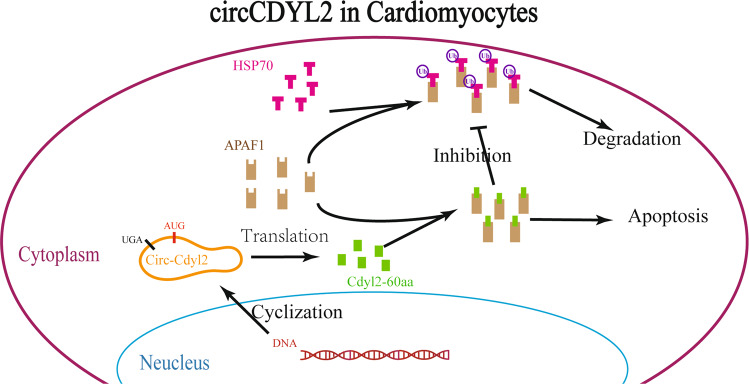
A graphical description of the mechanism. A schematic depicting that Cdyl2-60aa translated from circCDYL2 inhibits degradation by blocking APAF1 ubiquitination.

In conclusion, the above findings refine the current understanding of circRNAs and highlight that the role of polypeptides from circRNAs in HF is largely underestimated. We discovered that Cdyl2-60aa translated from circCDYL2 could accelerate the apoptosis of CMs and promote the development of HF in vivo and in vitro, suggesting that it could be a potential therapeutic target of HF in rats (Fig. 6). Although the coding ability of circCDYL2 was not conserved in other species, our future research will focus on the issue of whether Cdyl2-60aa works in humans.

## Supplementary information

supplementary information
