# Supplementary material for: Cdyl2-60aa encoded by CircCDYL2 accelerates cardiomyocyte death by blocking APAF1 ubiquitination in rats
Source: Exp Mol Med. 2023 Apr 3;55(4):860–9. doi: 10.1038/s12276-023-00983-5 (PMC10167378; doi:10.1038/s12276-023-00983-5)
Supplement: Supplementary file 1 — supplementary information [file 12276_2023_983_MOESM1_ESM.pdf]

Cdyl2-60aa Encoded by CircCDYL2 Accelerates Cardiomyocyte Death by Blocking APAF1 Ubiquitination in Rats

Yunfei Deng[1#], Xiaochen Zeng[2#], Yifei Lv[1], Zhiyuan Qian[1], Peijie Guo[3], Yi Liu[4], Shaoliang Chen[1]*

1 Department of Cardiology, Nanjing First Hospital, Nanjing Medical University, Nanjing, China.

2 Department of Clinical Laboratory, Children's Hospital of Nanjing Medical University, Nanjing, China

3 Department of Functional Examination, Nanjing First Hospital, Nanjing Medical University, Nanjing, China.

4 Department of Cell Biology, University of California, Davis, CA, USA.

**Correspondence to Shaoliang Chen, MD, PhD, Department of Cardiology, Nanjing First Hospital, Nanjing Medical University, Nanjing, China, NO.68 Changle Road, Nanjing, China, 210001. E-mail: chmengx@126.com*

#These authors contributed equally to this work

Running title: *Cdyl2-60aa promotes apoptosis via APAF1*

Supplementary Figure Legends

Supplementary Fig. 1 (***a***) Left: Echo images of hearts from Ctrl and HF groups; Right: Bar graph showing the LVEF in Ctrl and HF groups. (***b***) qRT-PCR analysis of circCDYL2 and linear Cdyl2 relative to β-actin in CMs. (***c***) qRT-PCR analysis of linear Cdyl2 in failing hearts and OGD treated NRCMs. (***d***) qRT-PCR analysis of circCDYL2 and linear Cdyl2 in NRCMs relative to fibroblast. (***e***) qRT-PCR analysis of circCDYL2 and linear Cdyl2 in NRCMs transfected with *si-circCDYL2*. (***f***) qRT-PCR analysis of circCDYL2 in NRCMs transfected with *ad-circCDYL2*. (***g***) Immunoblot analysis of autophagy- (P62 and LC3), proliferation- (Ki67) and pyroptosis-related (NLRP3, GSDMD and Caspase 1) proteins in NRCMs with overexpressed circCDYL2. (***h***) Bar graph showing the number of LC3-GFP dots in NRCMs transfected with *ad-circCDYL2*. *n=15, NS = not significant*. (***i***) Wound-healing analysis of NRCMs with overexpressed circCDYL2. *n=5, p = 0.866*. (***j***) CCK-8 analysis of NRCMs with overexpressed circCDYL2. *n=6, p = 0.108*. (***k***) Relative ATP production (Left) and ADP/ATP ratio (Right) in NRCMs with overexpressed circCDYL2. Data were expressed as mean ± s.d., *n=6*, *NS = not significant*. (***l***) Immunoblot analysis of autophagy- (P62 and LC3), pyroptosis- (NLRP3, GSDMD and Caspase 1) and proliferation- (Ki67) related proteins in hearts from NC and Ex groups. (***m***) Relative ATP production in hearts from NC and Ex groups. Data were expressed as mean ± s.d., *n=6*, *NS = not significant*. (***a***)(***b***)(***c***)(***d***)(***e***)(***f***) Data were expressed as mean ± s.d., *n=3*, * $p < 0.05$, *NS = not significant*.

Supplementary Fig. 2 IRES structure in circCDYL2. (***a***) Putative IRES sequences in circCDYL2. (***b***) Mutated IRES sequences in circCDYL2. (***c***) Putative sequence of amino acid from the ORF of circCDYL2.

Supplementary Fig. 3 (***a***)(***b***)(***c***) The conservation comparison of potential ORFs (***a***), IRESs (***b***) and possible polypeptides (***c***) in circCDYL2 from rat, mice (MMU_CIRCpedia_4128) and human (HSA_CIRCpedia_18832). Mutated nucleotides were highlighted with red color. (***d***) The secondary structures of IRESs predicted by *RNAfold* in MMU_CIRCpedia_4128 (Right) and HSA_CIRCpedia_18832 (Left). (***e***) Immunoblot analysis of possible endogenous Cdyl2-60aa in cardiomyocytes from rat, mice or human.

Supplementary Fig. 4 (***a***) Immunoblot of pyroptosis- (NLRP3, Caspase 1 and GSDMD), proliferation- (Ki67) and autophagy- (LC3 and P62) related proteins in NRCMs with overexpressed Cdyl2-60aa. (***b***) Quantification of LC3-GFP dots in NRCMs with overexpressed Cdyl2-60aa. Data were expressed as mean ± s.d., *n=15, two-tailed t-test, NS = not significant*. (***c***) Wound-healing analysis of NRCMs with overexpressed Cdyl2-60aa. *n=6, NS = not significant.* (***d***) CCK-8 analysis of NRCMs with overexpressed Cdyl2-60aa. *n=6, NS = not significant*. (***e***) Bar graphs showing the ATP (Right) and ADP/ATP ratio (Left) in NRCMs with Cdyl2-60aa overexpression. Data were expressed as mean ± s.d., *n=6*, *NS = not significant*. (***f***) FACS analysis of apoptotic NRCMs transfected with *circ-mut-ORF-His* or *circ-wt-ORF-His*. Data were expressed as mean ± s.d., *n=3*, * $p < 0.05$, *NS = not significant*. (***g***) Immunoblot analysis of Parp-1 and Caspase 3 (Cas 3) in NRCMs transfected with *circ-mut-ORF-His* or *circ-wt-ORF-His.* (***h***) Co-IP of HSC70 with APAF1 in NRCMs with overexpressed Cdyl2-60aa. (***i***) The schematic showing the structure of protein APAF1. (***j***) Upper: Gel analysis of PCR products (F1/R1) in *ad-circCDYL2* transfected HEK293 treated with RNase R; Lower: The results of Sanger sequence PCR products (F1/R1) in *ad-circCDYL2* transfected HEK293. (***k***) Immunoblot analysis of NOVA1 in OGD treated NRCMs. (***l***) Representative images of hearts sections by TTC staining (viable myocardium stained in red, and the infarcted areas appeared in pale). Dotted box showing the infarcted areas. Bar scales = 2 mm.

Supplementary Fig. 1

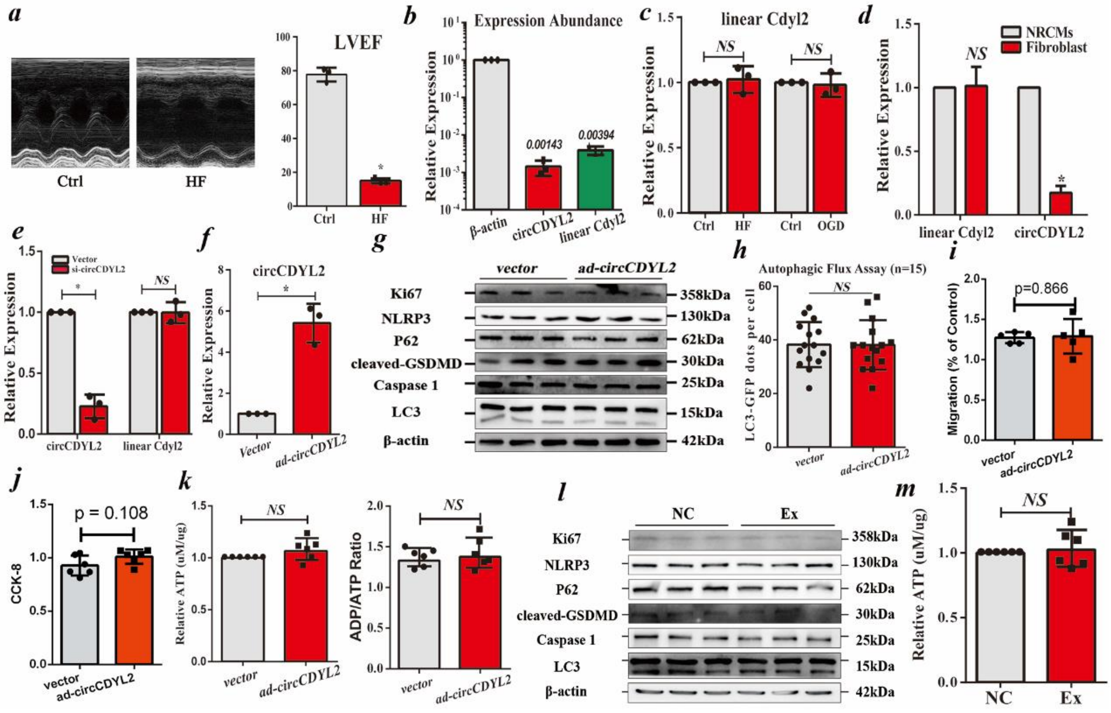

Supplementary Fig. 2

a

Rat_circ_Cdyl2 583nt wild type (ORF; IRES)

GUUGAAAGGAUUGUGGACAAGAGGAAGAACAAGA
AGGGGAAAUGGGAAUAUCUUAUCCGAUGGAAAGG
CUACGGCAGCACCGAGGACACGUGGGAGCCGGAG
CAUCACCUCCUGCACUGUGAGGAGUUUAUUGAUG
AGUUCAACGGGCUUCACUUGCCCAAGGAUAAGAA
GGUGAAGUCAGGGAAGCAGGCCGGAGCCUCCAAG
CUCCUUCGCGAUGCCCGCAGUCUGCCAGUGGAAA
GACUCUCCCAUCGACCACUGGAACCCGGGAAGAG
CAAAUCAACUUCCCACAAGCGGAAGCGCGUCAAC
UCUCCCCUGUCCAGAUCCAAGAAGGGGUCUUCAG
GAAAGGCUCCAGACAGGGCCACCAAGACCGUGUC
CUAUAGGACUACCCCCAGUGGCUUGCAAAUCAUG
CCCCUGAAGAAGGCUCAGAAUGGCUUGGAGAAUG
GAGAUGCUGGCUCAGAGAAGGAUGAAUCACACUU
UGGAAACGGGUCCCAUCAGCCAGAUUUGGAGUUG
AAUGACCAGCUUGGAGAGCAGGAGACCAGUGAUU
GCGACGGGACCCACUCUGCACUGGUGGAGAAUGG
AAUUG

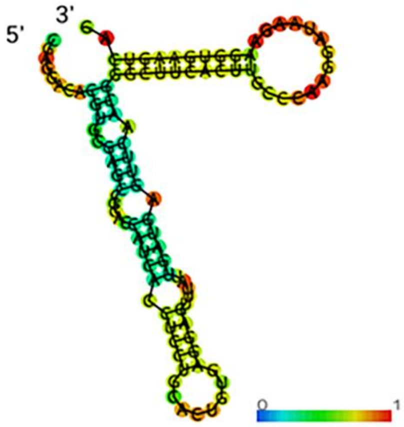

b

Rat_circ_Cdyl2 583nt mutant

GUUGAAAGGAUUGUGGACAAGAGGAAGAACAAGA
AGGGGAAAUGGGAAUAUCUUAUCCGAUGGAAAGG
CUACGGCAGCACCGAGGACAGUGGGGGACCGGAG
CUUCACCUAGUGCACUGUGAGGAGUUUAUUGAUG
AGUUCAACGUUGAACACUUGCCCAAGGAUAAGAA
GGUGAAGUCAGGGAAGCAGGCCGGAGCCUCCAAG
CUCCUUCGCGAUGCCCGCAGUCUGCCAGUGGAAA
GACUCUCCCAUCGACCACUGGAACCCGGGAAGAG
CAAAUCAACUUCCCACAAGCGGAAGCGCGUCAAC
UCUCCCCUGUCCAGAUCCAAGAAGGGGUCUUCAG
GAAAGGCUCCAGACAGGGCCACCAAGACCGUGUC
CUAUAGGACUACCCCCAGUGGCUUGCAAAUCAUG
CCCCUGAAGAAGGCUCAGAAUGGCUUGGAGAAUG
GAGAUGCUGGCUCAGAGAAGGAUGAAUCACACUU
UGGAAACGGGUCCCAUCAGCCAGAUUUGGAGUUG
AAUGACCAGCUUGGAGAGCAGGAGACCAGUGAUU
GCGACGGGACCCACUCUGCACUGGUGGAGAAUGG
AAUUG

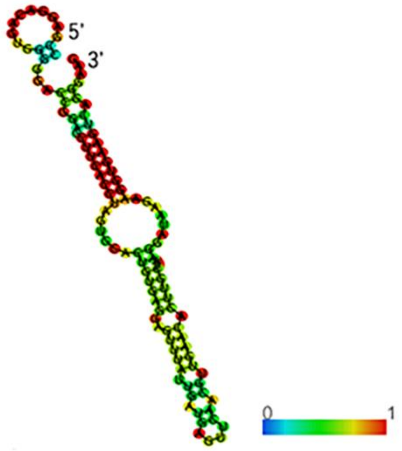

c

The sequence of Cdyl2-60aa

MPLKKAQNGLENGDAGSEKDESHFGNGSHQPDLELNDQLGEQETSDCDGTHSALVENGIG

Supplementary Fig. 3

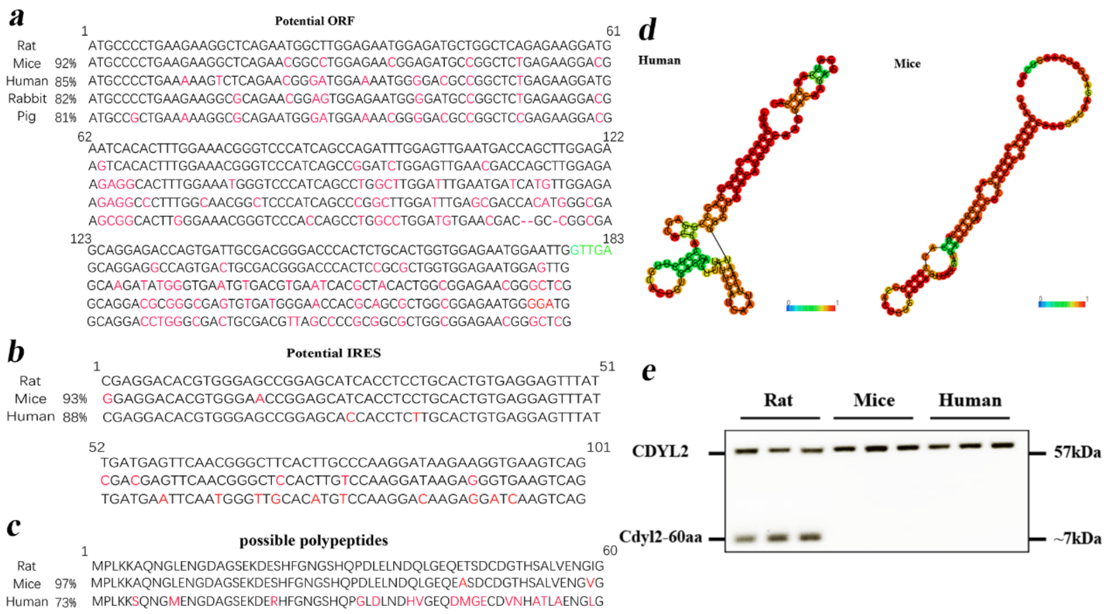

Supplementary Fig. 4

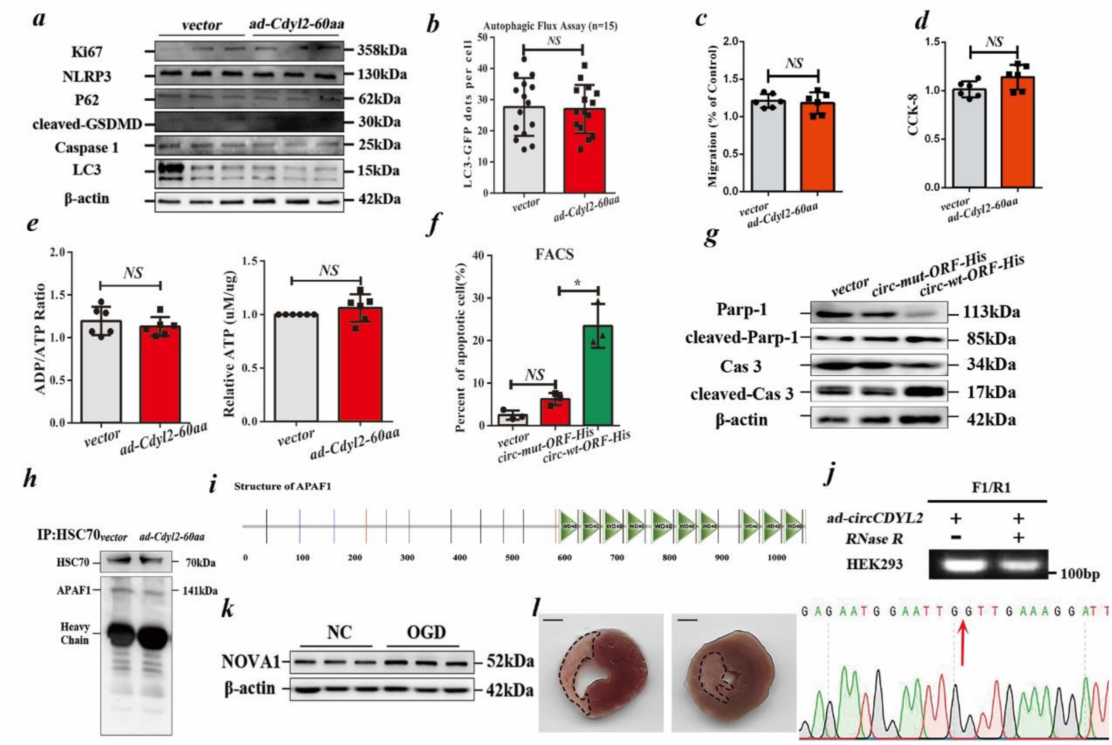

Supplementary Table 1

N	Unused	Total	%Cov	%Cov(50)	%Cov(95)	Accession
1	24.35	24.35	30.69	25.76	21.75	sp|P47198|RL22_RAT
2	22.71	22.71	25.060001	19.48	17.847	sp|P38983|RSSA_RAT
3	20.02	20.02	31.329999	29.7	26.13	sp|P60711|ACTB_RAT
4	19.89	19.89	30.329999	28.27	26.13	sp|Q62812|MYH9_RAT
5	6.17	6.17	7.564	5.641	4.615	sp|P63245|RACK1_RAT
5	0	6.17	7.7459998	6.338	6.338	tr|A0A0G2JZE6|A0A0G2JZE6_RAT
6	5.93	5.93	2.2569999	0.8892	0.684	tr|P68403|KPCB_RAT
6	0	5.93	2.11	0.8314	0.6395	sp|A0A0G2K5Q0|A0A0G2K5Q0_RAT
7	3.87	3.87	5.263	4.785	4.785	sp|Q6PDV7|RL10_RAT
8	3.37	3.37	5.5160001	4.385	1.98	sp|P84245|H33_RAT
9	2.33	2.33	6.329	4.008	4.008	sp|Q8VH46|AFAP1_RAT
10	0	2.33	20.900001	7.377	3.689	sp|D4AB98|AF1L1_RAT
10	2.3	2.3	23.39	8.257	4.128	sp|P62278|RS13_RAT
11	2.12	2.02	4.9910001	2.139	2.139	sp|P70550|RAB8B_RAT
11	0	2.02	4.9120001	2.105	2.105	sp|MORC99|RAB5A_RAT
12	2.11	2.01	17.5	6.875	6.875	sp|Q91V26|SPHK1_RAT
12	0	2.01	18.179999	7.143	7.143	tr|A0A0G2K373|A0A0G2K373_RAT
13	2	2	7.2290003	3.614	3.614	sp|Q4V7E8|LRRF2_RAT
13	1.98	1.98	7.5000003	3.75	3.75	sp|P27615|SCRB2_RAT
13	0	1.98	6.865	3.432	3.432	sp|Q07969|CD36_RAT
13	0	1.98	7.0749998	3.538	3.538	tr|A0A096MJ39|A0A096MJ39_RAT
13	0	1.98	6.1659999	4.021	4.021	tr|Q6IMX5|Q6IMX5_RAT
13	0	1.98	4.1510001	2.83	2.83	tr|A0A0G2JYC5|A0A0G2JYC5_RAT
13	0	1.98	5.7069998	3.722	3.722	tr|A0A096MIY7|A0A096MIY7_RAT
14	1.68	1.68	6.1730001	3.086	3.086	tr|Q9QX05|TLR4_RAT
14	0	1.68	6.2109999	3.106	3.106	tr|G3V7D8|G3V7D8_RAT
14	1.67	1.67	6.061	3.03	3.03	sp|Q64725|KSYK_RAT
14	0	1.67	12.2	12.2	12.2	tr|D3ZH44|D3ZH44_RAT
15	1.64	1.64	1.505	0.8467	0.8467	sp|F1LNJ1|F1LNJ1_RAT
16	1.59	1.59	1.191	0.536	0.536	sp|P19945|RLA0_RAT
16	0	1.59	1.194	0.5373	0.5373	tr|D3ZJT4|D3ZJT4_RAT

Name	Species	Peptides(95%)
60S ribosomal protein L22 OS=Rattus norvegicus GN=Rpl22 PE=1 S	Rattus norveg	13
40S ribosomal protein SA OS=Rattus norvegicus GN=Rpsa PE=1 SV	Rattus norveg	9
Actin, cytoplasmic 1 OS=Rattus norvegicus GN=Actb PE=1 SV=1	Rattus norveg	12
Myosin-9 OS=Rattus norvegicus GN=Myh9 PE=1 SV=2	Rattus norveg	11
Receptor of activated protein C kinase 1 OS=Rattus norvegicus	Rattus norveg	4
Guanine nucleotide-binding protein subunit beta-2-like 1 OS=Ra	Rattus norveg	4
Protein kinase C beta type OS=Rattus norvegicus GN=Prkcb PE=1	Rattus norveg	3
Protein kinase C OS=Rattus norvegicus GN=Prkcb PE=1 SV=2	Rattus norveg	3
60S ribosomal protein L10 OS=Rattus norvegicus GN=Rpl10 PE=1 S	Rattus norveg	2
Histone H3.3 OS=Rattus norvegicus GN=H3-3b PE=1 SV=1	Rattus norveg	1
Actin filament-associated protein 1 OS=Rattus norvegicus GN=Af	Rattus norveg	2
Actin filament-associated protein 1-like 1 OS=Rattus norvegicu	Rattus norveg	1
40S ribosomal protein S13 OS=Rattus norvegicus GN=Rps13 PE=1 S	Rattus norveg	2
Ras-related protein Rab-8B OS=Rattus norvegicus GN=Rab8b PE=1	Rattus norveg	1
Ras-related protein Rab-5A OS=Rattus norvegicus GN=Rab5a PE=1	Rattus norveg	1
Sphingosine kinase 1 OS=Rattus norvegicus GN=Sphk1 PE=1 SV=1	Rattus norveg	3
Sphingosine kinase 1 OS=Rattus norvegicus GN=Sphk1 PE=1 SV=1	Rattus norveg	1
Leucine-rich repeat flightless-interacting protein 2 OS=Rattus	Rattus norveg	1
Lysosome membrane protein 2 OS=Rattus norvegicus GN=Scarb2 PE=	Rattus norveg	2
Platelet glycoprotein 4 OS=Rattus norvegicus GN=Cd36 PE=1 SV=1	Rattus norveg	1
Glycoprotein IIIb OS=Rattus norvegicus GN=Cd36 PE=1 SV=1	Rattus norveg	1
Glycoprotein IIIb OS=Rattus norvegicus GN=Cd36 PE=1 SV=2	Rattus norveg	1
Glycoprotein IIIb OS=Rattus norvegicus GN=Cd36 PE=1 SV=1	Rattus norveg	1
Glycoprotein IIIb OS=Rattus norvegicus GN=Cd36 PE=1 SV=1	Rattus norveg	1
Toll-like receptor 4 OS=Rattus norvegicus GN=Tlr4 PE=1 SV=1	Rattus norveg	3
Toll-like receptor 4 OS=Rattus norvegicus GN=Tlr4 PE=1 SV=1	Rattus norveg	1
Tyrosine-protein kinase SYK OS=Rattus norvegicus GN=Syk PE=1 S	Rattus norveg	1
Tyrosine-protein kinase OS=Rattus norvegicus GN=Syk PE=1 SV=1	Rattus norveg	1
Non-specific serine/threonine protein kinase OS=Rattus norvegi	Rattus norveg	2
60S acidic ribosomal protein P0 OS=Rattus norvegicus GN=Rplp0	Rattus norveg	1
60S acidic ribosomal protein P0 OS=Rattus norvegicus GN=Rplp01	Rattus norveg	1

Supplementary Table 2

antibody	FI-NC						FI-EX					
14-3-3 theta/tau (Ab-232)	1.11	0.96	0.96	0.93	0.93	0.87	1.01	1.04	0.91	0.89	1.11	1.04
14-3-3 theta/tau (Phospho-Ser232)	1.11	1.02	1.01	0.99	0.92	0.94	0.9	0.94	0.87	0.93	1.1	0.97
14-3-3 zeta (Phospho-Ser58)	0.99	1.05	0.91	0.95	1	1.11	1.06	0.98	0.92	1.04	1.08	1.04
14-3-3 zeta (Ab-58)	1.08	0.97	0.97	1.01	0.98	0.98	1.12	0.95	1.1	0.97	0.96	0.92
14-3-3 zeta/delta (Ab-23	0.96	1.09	0.9	1.07	0.94	0.93	1.01	1.06	1.03	0.96	1	1.04
14-3-3 zeta/delta (Phosp	1.01	0.88	1.06	0.95	1.11	1.11	1.12	0.9	1.11	1.02	1.03	1.03
AKT (Ab-308)	0.93	0.92	0.91	0.9	1.08	1.09	1.31	0.99	1.23	1.11	1.42	1.29
AKT (Ab-473)	1.09	0.91	0.89	1.02	0.96	1.11	0.87	1.06	0.87	0.88	1.06	1
AKT (N-term)	0.96	0.95	1.04	1.11	1.05	0.87	0.92	0.93	0.98	0.87	0.89	1.08
AKT (Phospho-Ser473)	1.02	0.94	1.06	1.03	1.01	0.87	0.94	1.06	0.96	1.07	0.99	0.94
AKT (Phospho-Thr308)	0.94	1.03	1.05	0.99	0.9	0.97	0.87	0.93	0.93	1.09	1.09	1.01
AKT1 (Ab-474)	0.94	1.04	0.95	1.06	1.05	1.08	0.96	0.99	0.87	1.06	0.98	1.06
AKT1 (Ab-72)	0.96	0.96	0.93	0.94	0.9	0.9	1.03	1.09	1	1.03	1.07	1.01
AKT1 (Phospho-Thr72)	0.92	0.97	1.06	0.9	1.08	1.03	1.07	1	1.01	0.93	1.02	0.91
AKT1 (Phospho-Tyr474)	0.9	1.1	1.1	1.08	0.88	0.99	0.94	0.95	0.98	0.98	0.89	0.97
AKT2 (Ab-474)	0.92	1.09	1.05	0.96	1.03	1.09	0.94	0.91	0.89	0.89	1.09	0.89
AKT2 (Phospho-Ser474)	1	0.96	1.03	1.07	0.97	1.08	0.97	0.88	1.01	0.97	0.94	1.01
APAF-1-ALT (C-term)	0.92	0.96	0.96	0.87	0.92	1.05	1.39	1.19	1.52	1.06	1.34	1.72
A-RAF (Ab-301/302)	1.02	0.91	0.93	0.94	1.12	0.88	1.03	0.9	1.01	1.06	0.95	1.03
A-RAF (Phospho-Tyr301	1	0.88	0.9	1	1.12	0.9	0.99	0.87	1.05	1.07	1	0.99
ARF4 (inter)	1.07	0.89	0.9	1.03	1.09	0.88	1.11	1.02	1.07	1.04	0.93	0.97
ASK1 (Ab-83)	1.09	0.98	0.92	0.88	0.99	1.02	1.01	0.95	1.12	0.94	1.03	0.89
ASK1 (Ab-966)	0.9	1.01	0.91	1.01	1.12	1.07	1	1.07	0.87	0.9	1.08	1.08
ASK1 (Phospho-Ser83)	0.89	1.02	1.07	1.02	0.89	0.98	0.99	0.93	1.1	1.01	0.96	1.11
ASK1 (Phospho-Ser966)	0.9	1.12	0.92	0.91	0.95	1.03	1.12	1.02	1.02	0.9	1.05	0.94
ATM (Ab-1981)	1.06	0.89	0.9	1.01	1.11	1.03	0.9	0.9	0.95	1.07	0.89	1
ATRIP (Ab-68/72)	0.95	0.95	0.97	1.06	1	0.97	0.93	0.91	1.05	0.89	1.02	1.01
ATRIP (Phospho-Ser68/72)	1.12	1.02	1.03	0.9	0.98	1.04	1.05	0.92	0.95	1.07	1.11	0.95
BAD (Ab-112)	1.03	1.04	0.95	1.08	1.05	0.98	1.07	1.11	0.97	0.91	1.02	0.93
BAD (Ab-134)	1.01	0.92	1.03	0.89	0.98	0.93	0.94	0.91	1.02	1.09	0.96	0.96
BAD (Ab-136)	0.9	0.91	0.91	1.08	1.02	1.12	1.05	0.9	1.04	0.91	0.95	0.97
BAD (Ab-155)	1.01	1.06	0.91	1.1	1.05	0.9	0.94	1.1	1.11	1.04	1.1	0.95
BAD (Ab-91/128)	0.87	0.87	1.03	0.87	1.03	1.1	0.94	0.87	0.91	1.08	0.96	0.95
BAD (Phospho-Ser112)	1.1	0.98	1.03	1.06	1.11	0.93	0.95	1.06	0.96	0.95	1.09	0.95
BAD (Phospho-Ser134)	1.02	1.12	1.07	0.92	1.05	1.04	1.03	1.02	1.08	0.89	1.07	1.04
BAD (Phospho-Ser136)	1.01	0.92	1.09	1.08	1.05	1	0.9	1.11	0.97	1.04	1.03	0.88
BAD (Phospho-Ser155)	1.02	1.06	0.94	0.92	1.07	1.07	0.96	0.88	0.97	1.05	1.12	1.1
BAD (Phospho-Ser91/12	0.93	1	0.97	1.05	0.91	0.99	0.93	0.97	0.96	1.05	0.87	0.94
Bak (N-term)	0.93	0.94	1.1	1.01	0.9	0.88	1.06	1.09	0.93	1.1	0.92	1.02
Bax (N-term)	0.91	0.92	0.89	0.96	0.93	1.02	1.02	0.89	1.03	1.02	1.03	1.02
BCL-2 (Ab-56)	0.95	0.87	1.06	0.94	0.87	0.92	1.04	0.9	0.98	1.12	1.08	1.01
BCL-2 (Ab-69)	0.9	1.02	1.09	0.99	0.92	0.92	0.89	1	1.02	0.96	1.05	0.93
BCL-2 (Ab-70)	0.87	1.11	1.02	1.07	1.1	0.96	1.07	1.09	0.97	0.95	0.88	1.05
BCL-2 (Phospho-Ser70)	0.89	1.02	0.98	1.08	0.97	0.89	0.91	1.09	0.92	1.12	1.03	1.09
BCL-2 (Phospho-Ser87)	0.95	0.99	0.99	0.94	1.12	0.91	0.94	0.97	1.03	0.88	1.02	1.04
BCL-2 (Phospho-Thr56)	1.05	1.08	0.93	1.03	1.01	1.06	1.11	0.94	0.94	0.93	0.91	1.11
BCL-2 (Phospho-Thr69)	0.87	0.95	1	0.99	1.03	0.93	0.91	1.09	1.12	1.01	0.92	0.96
BCL-XL (Ab-47)	1.09	0.92	0.9	0.95	1.01	1.02	0.97	0.99	1.11	1.06	0.92	0.87
BCL-XL (Ab-62)	0.91	1.05	0.89	1.1	1.03	0.97	1.07	0.95	1.09	0.97	1.1	1.06
BCL-XL (Phospho-	0.99	1.09	1.1	0.98	0.88	0.88	1.06	1.11	1.09	1.01	0.9	0.92
BCL-XL (Phospho-Thr47	1.04	1.1	0.88	0.9	1.04	1.1	0.93	1.07	1.07	1.08	0.92	0.97
BID (Ab-78)	1.07	1.04	0.98	0.95	1.08	0.89	1.02	0.99	0.98	1.08	1.06	1.09

BID (Phospho-Ser78)	0.93	1.08	0.89	1.08	1.07	1.07	1.05	0.9	1	0.98	0.89	0.99
BIM (Ab-69/65)	0.94	1.1	0.92	1.08	1.07	0.89	0.92	1.12	0.98	1.11	0.89	0.89
BIM (Phospho-	1.12	0.96	1.03	1	1.04	1.08	0.95	0.91	1.08	0.97	0.98	1.06
B-RAF (Ab-446)	0.95	1.05	0.88	0.91	1.03	0.93	1.05	0.94	0.93	0.88	1	1.02
B-RAF (Ab-598)	1.05	1.11	0.94	1.05	0.99	0.91	1.04	0.92	0.97	1.11	1.03	0.88
B-RAF (Ab-601)	1	1.09	1.03	1.03	0.93	0.98	1.03	1.03	1.07	0.94	1.03	1
B-RAF (Phospho-	0.99	0.95	1.04	0.91	1.09	0.91	1.07	0.95	0.9	0.88	1.11	1.07
B-RAF (Phospho-Ser601)	1.1	0.94	0.87	0.95	1.08	0.91	0.94	1	1	1.07	0.9	0.92
B-RAF (Phospho-Thr598	1.01	1.12	0.9	1.06	1	1.05	0.93	0.89	1.02	1.06	1.1	0.95
CaMKII (Ab-286)	0.95	0.93	0.88	0.9	0.98	1.09	0.92	0.97	0.97	0.93	1.12	1.03
CaMKII (Phospho-Thr28	1.05	1.05	0.92	0.99	1.12	0.96	1	0.97	1.09	0.92	1.11	0.89
CASP6 (Ab-Ser257)	1	1.08	0.89	0.95	1.04	0.95	0.87	1.06	0.95	1.05	1.05	1.12
CASP6 (Phospho-Ser257)	0.88	1.09	0.88	1.01	0.9	0.99	1	0.97	1.02	0.87	0.96	1.07
CASP8 (Ab-347)	0.87	1.1	1.03	1.06	1.06	0.94	0.92	1.12	0.97	1	0.89	1.11
CASP8 (Phospho-Ser347)	1.01	1	0.96	0.95	1.03	0.95	0.92	0.97	1.02	0.98	0.9	0.92
CASP9 (Ab-125)	1.01	1	0.87	1.08	1.09	0.89	0.89	1.05	1.05	0.92	0.96	1.01
CASP9 (Phospho-Thr125)	1.07	1.04	0.93	0.97	0.92	1.02	0.88	0.92	0.9	0.9	1.02	0.91
Caspase 10 (C-term)	0.9	1.09	0.92	0.88	0.9	0.97	0.87	0.9	1.01	1.04	1	0.95
Caspase 9 (Ab-144)	1.01	1.11	1.11	1.11	1.08	1.06	0.88	1.08	0.88	0.92	0.99	1.03
Caspase 9 (Ab-153)	0.99	0.98	0.99	1.12	0.97	0.99	0.89	1.03	0.89	1.02	1.1	1.06
Caspase 9 (Ab-196)	1.01	1.06	0.9	0.99	1.06	1.03	1.02	0.98	0.97	0.9	0.96	1.06
Caspase 9 (Phospho-Ser1	1.12	0.93	0.91	0.98	0.93	1.11	0.93	0.93	0.87	1.01	1.11	1.03
Caspase 9 (Phospho-Ser1	0.92	1.04	0.94	1.08	1.04	0.96	0.99	1.02	1.09	0.91	0.94	0.97
Caspase 9 (Phospho-Tyr153)	1.04	1.01	0.98	1.06	0.98	1.06	1.02	1.04	1.01	0.99	0.95	0.9
Caspase-3 (Ab-150)	1.09	1.04	1.12	1.08	0.98	1.01	0.9	0.88	1.03	0.97	0.88	1.01
Caspase-3 (Phospho-Ser1	1.09	1.1	0.87	1.12	0.98	1.07	1.09	1.05	0.93	1.02	0.9	1.04
CDC2 (Ab-15)	0.87	0.94	0.97	0.92	1	0.92	1.06	1.1	0.89	0.9	1.08	0.91
CDC2 (Phospho-Tyr15)	0.92	1.11	0.91	0.96	1.02	1.08	1.06	0.88	0.88	1	1.07	0.9
CDK1/CDC2 (Ab-14)	1.04	0.99	1.02	1.07	1.11	0.94	0.99	0.92	0.97	0.9	1.05	0.96
CDK1/CDC2 (Phospho-Tl	1.06	1.07	1.05	1.07	0.89	0.87	1.11	1.03	0.99	1.06	0.87	0.97
Chk1 (Ab-280)	1	1.02	1.11	0.88	0.91	1.05	1.12	1.08	1.03	1.08	0.99	1.01
Chk1 (Ab-286)	1.03	0.91	0.98	1.07	0.96	1.11	0.9	0.91	0.88	0.96	1.03	0.94
Chk1 (Ab-317)	0.92	1.01	1.09	0.95	1.06	0.87	1.08	0.99	0.87	1.07	0.92	0.87
Chk1 (Ab-345)	0.88	0.92	1.03	1.09	1.04	0.95	1	0.98	1.08	0.99	1.05	1.05
Chk1 (Phospho-Ser280)	0.99	1.12	1.01	1	1	1.05	0.91	1.08	0.89	0.96	1.1	1.1
Chk1 (Phospho-Ser286)	0.91	1.02	1.12	0.91	0.87	1.08	0.94	0.93	1.01	1.03	1.11	0.9
Chk1 (Phospho-Ser317)	0.95	0.93	0.89	1.06	0.97	1.1	0.89	1.01	0.89	1.1	1.08	0.96
Chk1 (Phospho-Ser345)	0.95	0.91	1.01	0.96	0.98	1.11	0.99	0.98	1.01	0.92	1.03	1.06
Chk2 (Ab-383)	0.88	0.96	1.03	1.1	0.98	1.06	1.11	1.12	1.09	1.1	0.88	0.99
Chk2 (Ab-387)	1.01	0.99	1.05	0.95	0.89	0.91	1.07	0.95	0.87	0.93	1.09	0.95
Chk2 (Ab-516)	1.1	1.01	0.98	1.01	1.11	1	0.89	1.11	1.08	0.96	0.91	1.07
Chk2 (Ab-68)	1.01	1.08	1.09	0.98	0.89	1.12	1.02	1.07	0.99	0.97	0.87	0.93
Chk2 (Phospho-Ser516)	0.97	0.92	1	0.9	0.87	1.11	0.87	1.04	0.97	0.98	0.98	0.99
Chk2 (Phospho-Thr383)	1.02	1.06	1.03	0.91	0.96	0.98	1.11	0.92	0.98	1.02	0.88	1.05
Chk2 (Phospho-Thr387)	1.01	0.96	0.88	0.97	1.07	0.89	1.12	0.99	0.88	0.96	1.02	1.06
Chk2 (Phospho-Thr68)	0.91	0.94	0.9	0.9	1.12	1.07	0.97	1	0.89	1.03	1.11	1
c-Jun (Ab-243)	0.94	1.12	0.94	1.11	0.98	0.94	1.08	1.02	1.11	1.08	0.9	0.92
c-Jun (Ab-91)	1.05	1.01	0.96	0.97	1.04	0.91	0.95	1.04	0.95	0.98	0.97	0.93
c-Jun (Phospho-Ser243)	0.93	0.91	0.91	0.91	1.03	1.09	1.09	0.99	1.05	1.09	0.99	0.93
c-Jun (Phospho-Thr91)	0.99	0.92	1.01	0.89	1.04	1.06	1.08	0.97	0.87	1.07	0.88	1.03
Cytochrome c (inter)	0.91	1.06	1.11	0.94	1.05	1.06	1.06	1.05	1.08	0.87	0.92	1.1
DAXX (Ab-668)	0.98	0.94	0.97	0.9	1	0.87	1.05	0.96	0.91	1.11	0.97	0.97
DAXX (inter)	1	1.1	1.03	1.05	0.91	1.08	1.02	0.92	1.1	0.98	0.89	1.11
DAXX (Phospho-Ser668)	0.93	1.04	1.05	1.09	1.05	0.95	1.05	1.12	1.06	1.08	0.92	1.09
ERK1/2 (N-term)	1.03	1.08	1.08	0.9	1.08	1.05	1.08	0.96	0.94	0.97	1.05	0.93
ERK1-p44/42 MAP Kinas	0.92	0.95	0.91	1.11	0.89	1.09	1.03	1.12	0.89	0.99	0.91	1.02

ERK1-p44/42 MAP Kinas	0.98	1.08	0.97	0.98	0.87	1.04	0.96	1.08	1.05	0.88	0.87	1.04
ERK1-p44/42 MAP Kinas	0.89	0.96	1.07	0.94	1.11	0.93	0.98	0.9	1.1	0.94	0.93	0.88
ERK1-p44/42 MAP Kinas	0.92	1.12	0.95	1.04	1.12	1.1	0.9	0.99	0.88	0.93	1	1.12
FADD (Ab-194)	1.05	1.06	1.12	1.09	1.11	1.09	1.06	0.88	0.98	1.01	1.06	1.12
FADD (Phospho-Ser194)	0.88	0.95	0.93	1.03	0.89	0.95	1.01	1.01	1.11	1.07	0.9	0.95
Fas (C-term)	0.95	1.05	1.07	1.01	0.98	0.98	1.1	0.91	1.01	0.93	1.06	1.01
FAS ligand (inter)	0.87	0.88	1.06	0.97	1.03	1.09	0.98	1.12	1.09	1.05	1.08	1.04
FKHR (Ab-256)	0.89	0.95	0.99	0.95	1.03	0.99	1.09	1.05	1.07	1.11	0.92	1.1
FKHR (Ab-319)	0.96	1.02	1.06	1.1	0.88	1.06	1.08	1.07	0.87	0.89	1.11	0.97
FKHR (Phospho-Ser256)	0.95	0.96	1.03	1.09	1.02	0.87	1.03	1.04	0.93	0.99	1.05	0.92
FKHR (Phospho-Ser319)	1	1.01	0.92	1.07	0.94	1.09	0.91	0.89	1.07	1.07	1.05	0.91
FOXO1/3/4-PAN (Ab-24	1	0.96	1.05	0.9	1.01	1.07	0.94	0.91	1.09	1.1	0.97	0.88
FOXO1/3/4-PAN (Phosp	0.93	0.99	0.99	1.06	0.93	1.12	0.92	0.98	1.01	0.99	0.89	1.02
FOXO1A (Ab-329)	1	1.11	1.05	1.08	0.95	1.1	1.01	0.97	0.98	0.95	0.88	1.05
FOXO1A (Phospho-Ser32	1.02	0.94	1.04	1.07	0.94	0.94	0.91	1.05	1.05	0.87	0.87	0.95
HSP 90-beta (Ab-226)	1.01	0.95	0.94	0.9	1	0.91	1.1	1.09	1.07	1.02	0.93	1.09
HSP 90-beta (Phospho-Se	0.98	1.12	1.06	0.95	1.08	1.09	1.05	1.11	0.96	0.95	1.03	1.04
HSP27 (Ab-15)	0.88	1.12	0.87	0.94	0.95	1.12	1.01	1.12	1.08	1.09	0.89	1.06
HSP27 (Ab-78)	1.04	1.12	1.11	0.96	0.96	1.1	1.03	0.89	1.07	0.93	1.02	1.01
HSP27 (Ab-82)	1.03	0.9	1.02	0.9	0.97	1.08	0.92	1.1	0.95	1.01	0.95	1.09
HSP27 (Phospho-Ser15)	1.03	1.06	1.12	1.1	0.99	0.95	0.98	1.05	0.89	0.97	1.06	1.12
HSP27 (Phospho-Ser78)	1.08	1.01	0.9	0.99	1.1	0.94	1.07	0.9	1.07	0.99	1.02	0.95
HSP27 (Phospho-Ser82)	0.87	0.9	0.94	1.01	0.92	1.1	0.93	1.11	1.05	1.11	0.99	0.92
HSP90A (C-term)	0.87	1.07	0.93	0.92	1.04	1.07	1.01	1.06	0.89	0.89	0.93	0.87
HSP90B (Ab-254)	1.05	1	1.06	0.89	0.91	1.02	1.12	0.87	0.9	0.94	0.99	0.97
HSP90B (Phospho-Ser254)	1.01	0.88	1.11	0.92	1.07	0.92	0.96	1.11	1.04	1.12	0.9	1.02
IkB-alpha (Ab-32/36)	1.03	1	0.97	1.11	1.08	1.09	1.04	1.03	1.12	1.12	0.91	0.98
IkB-alpha (Ab-42)	1.04	1.1	1.04	1.11	0.91	1.06	1.07	1.11	1.02	0.93	1.1	1.1
IkB-alpha (Phospho-Ser3	1.01	1.12	1.06	1	1.08	0.94	0.96	0.91	0.88	0.94	1.03	1.11
IkB-alpha (Phospho-Tyr4	0.97	0.97	1.02	1.04	0.99	1.08	0.97	0.9	0.98	0.94	0.88	1.09
IkB-beta (Ab-19)	0.91	1.01	0.96	0.96	0.94	1.04	1.04	1.11	0.91	1.04	1.02	0.94
IkB-beta (Phospho-Thr1	1.07	0.87	0.94	0.97	1	0.9	1.12	1.07	1.08	0.9	0.93	1.03
IkB-epsilon (Ab-22)	0.88	0.92	0.99	1.04	1.08	1.02	1.05	1.08	0.9	1.07	0.92	1.03
IkB-epsilon (Phospho-Ser22)	1.07	1.04	1.12	0.87	0.94	0.96	1.09	1.09	0.94	1.03	0.97	1
IKK alpha (Ab-23)	1	1.11	0.98	1.1	0.94	0.98	0.9	0.98	1.04	0.96	0.92	0.94
IKK alpha (Phospho-Thr2	0.99	1.02	1.11	1.06	0.94	0.88	1.04	0.98	0.92	1.08	1.02	1.12
IKK beta (Ab-188)	1.05	1.01	1	0.98	0.96	1.05	1.12	1.05	1.02	1.03	1.07	1.09
IKK beta (Ab-199)	0.98	1.02	0.87	0.93	0.98	0.95	1	0.9	0.89	0.92	1.12	1.11
IKK beta (Phospho-Tyr18	1.04	1.12	0.89	1.05	0.9	1.07	0.87	1.11	1.02	1.1	1.03	1.04
IKK beta (Phospho-Tyr19	0.95	1.1	0.96	1	1.07	1.08	1.09	1.02	1	1	0.92	0.92
IKK gamma (Ab-85)	0.89	1.01	1.05	1.06	0.97	0.98	0.93	0.88	1.03	1.02	0.94	1.05
IKK gamma (Phospho-Se	1.1	0.9	0.97	1.01	0.88	1.06	0.96	0.92	0.97	0.99	0.98	1.11
IKKa/b (AB-180/181)	1.02	1.1	1.12	1.06	0.9	0.95	1.05	0.9	0.95	0.89	0.89	0.95
IKKa/b (Phospho-Ser180	1.03	1.02	0.93	0.95	1.1	1.01	1.09	1.12	1.03	0.89	0.88	1.09
IKK-GAMMA (Ab-31)	1	1.12	0.95	1.09	1.05	0.9	1.05	1.06	0.91	1.06	0.96	1.02
IKK-GAMMA (Phospho-S	0.91	0.9	1.06	1.08	1.02	1	1.07	1.1	1.04	0.87	0.96	0.94
JNK1/2/3 (Ab-183)	1.12	0.99	0.96	0.92	1.09	0.97	1.09	0.96	0.96	0.88	1.03	0.87
JNK1/2/3 (Phospho-Thr1	0.88	0.94	0.94	0.91	1.11	1.08	0.94	1	1.07	1.11	0.99	1.1
Lamin A/B(lamin A/C) (A	1.11	0.97	1.1	0.94	0.97	1.09	1.02	0.98	1.02	1.06	1.12	0.99
Lamin A/B(lamin A/C) (I	1.11	0.94	1.07	0.96	1.07	0.87	1.11	1.07	0.88	1.06	0.96	0.9
Lamin A/C (Ab-22)	1	1.09	0.88	0.87	1.01	1.09	0.91	0.89	1.01	1.1	0.91	1.03
Lamin A/C (Phospho-Ser22)	1.05	0.99	0.94	1.07	0.97	1	1.11	1.12	1.05	0.9	0.93	0.91
MAP3K7/TAK1 (Ab-	0.98	1.06	0.95	1.09	0.99	0.87	0.94	1.04	0.96	1.07	1.06	0.97
MAP3K7/TAK1 (Ab-439))	0.96	1.01	1.01	0.95	1.04	1.04	1.02	1.02	0.93	1.09	1.05	1.04

MKK7/MAP2K7 (Ab-	0.87	0.92	0.87	1.07	0.9	0.96	0.99	0.94	1.02	0.93	0.89	1.09
MKK7/MAP2K7 (Phospho-Ser271)	1	1.11	0.9	1	0.98	0.88	0.95	0.94	1.05	0.87	0.95	0.99
MKK7/MAP2K7 (Phospho-Thr275)	0.97	0.91	1.08	1	0.93	1	1.09	1.04	1.04	1.09	0.94	1.06
NFkB-p100 (Phospho-Se	0.93	0.89	0.87	1.09	1	0.89	0.89	0.95	0.97	1.08	0.91	0.94
NFkB-p100/p52 (Ab-865)	1.1	0.92	0.96	1	1.04	1.03	0.97	0.97	1.08	1.04	0.98	0.9
NFkB-p100/p52 (Ab-869)	0.93	1.01	1.02	0.99	1.11	1.12	1.12	0.96	1.02	1.05	1.05	1
NFkB-p100/p52 (Phospho-Ser865)	1.05	1.09	1.02	1	0.93	0.88	0.92	1.03	0.95	1.08	0.87	1
NFkB-p100/p52 (Phospho-Ser869)	0.89	1.1	1.06	0.91	1.08	1.03	1	1	0.87	1.1	0.93	1.11
NFkB-p105 (Ab-927)	1.06	0.87	0.93	1.11	0.87	1	0.87	0.98	0.94	1.01	1.01	1.1
NFkB-p105 (Phospho-Se	1.05	0.92	1.06	0.92	1.05	1.1	0.87	1.07	0.88	0.9	0.94	0.93
NFkB-p105/p50 (Ab-337)	1.08	0.95	1.02	0.95	1.09	0.87	1.12	0.88	1.05	0.95	0.92	1.01
NFkB-p105/p50 (Ab-893)	0.96	0.88	0.88	1.1	1.02	1.01	1.11	0.91	1.09	0.92	1.05	0.93
NFkB-p105/p50 (Ab-907)	0.98	0.93	0.99	0.96	0.91	0.91	0.96	0.95	1.08	0.93	0.96	1.01
NFkB-p105/p50 (Ab-932	0.94	1.04	0.94	0.94	0.97	1.06	1.09	1.12	0.92	1.03	1.03	1.09
NFkB-p105/p50 (Phospho-Ser337)	0.98	0.98	1.12	1.11	1.02	0.91	1.03	0.92	0.96	0.97	1.11	1.06
NFkB-p105/p50 (Phospho-Ser893)	1.04	1.03	0.91	1.04	0.88	0.88	0.97	0.93	1.11	0.89	0.91	1.03
NFkB-p105/p50 (Phospho-Ser907)	1.11	0.87	1.02	1.05	0.99	0.96	1.06	1.11	1	0.97	0.92	0.92
NFkB-p105/p50 (Phosph	1.08	1.1	0.96	0.94	1.08	0.98	0.94	0.97	1.12	1.05	0.97	1.04
NFkB-p65 (Ab-254)	0.97	1.06	1.07	0.9	1.11	1.03	0.91	0.93	0.92	0.9	0.92	0.98
NFkB-p65 (Ab-276)	1.02	0.91	1.09	1.07	0.91	0.99	0.89	0.96	1.03	0.98	1.04	0.97
NFkB-p65 (Ab-311)	1.03	0.92	1.09	1.1	0.96	0.96	1.03	0.9	0.87	1.12	0.95	0.88
NFkB-p65 (Ab-468)	1.03	1.08	1.03	1.04	1.06	0.96	1.05	0.93	0.88	1.11	0.93	0.98
NFkB-p65 (Ab-529)	0.9	0.99	1.1	0.88	1	1.01	1.1	0.95	0.93	0.9	1	1.1
NFkB-p65 (Ab-536)	0.9	0.98	1.08	0.96	1.01	0.95	0.92	0.94	1.12	0.94	1.12	1.06
NFkB-p65 (Phospho-Ser276)	1.01	1.05	0.99	1.06	0.92	0.87	1.1	1.11	1.07	1.02	0.92	1
NFkB-p65 (Phospho-Ser311)	0.91	1.12	0.9	0.94	1.07	1.12	0.99	1.11	0.93	0.97	1.06	0.92
NFkB-p65 (Phospho-Ser468)	1.1	1.01	1.02	1.01	1.08	0.92	0.88	1.12	0.92	0.91	0.97	0.97
NFkB-p65 (Phospho-Ser529)	0.9	0.97	0.99	1.08	0.89	1.05	1.04	0.9	0.9	0.89	0.91	0.88
NFkB-p65 (Phospho-Ser536)	1.01	0.93	1.09	1.05	1.05	0.9	1.11	1.11	0.9	0.97	0.96	1.08
NFkB-p65 (Phospho-Thr254)	1.04	0.91	1	1.09	0.99	1.03	0.95	1	0.98	1.06	1.02	1.05
p53 (Ab-37)	1.04	1.04	1.11	0.91	0.88	0.95	0.99	1.12	0.94	0.97	0.97	1.04
p53 (Ab-392)	0.97	1.02	0.88	1.12	0.94	0.89	1.04	1.12	0.97	1.11	1.08	0.92
p53 (Ab-6)	0.93	0.89	0.96	0.96	0.91	0.97	1.02	1.05	1.11	0.89	0.91	0.91
p53 (inter)	0.99	0.9	1.11	1.12	0.9	1.06	1.11	0.94	0.89	1.11	0.94	1.11
p53 (Phospho-Ser37)	1.08	0.98	0.97	0.92	0.99	0.93	1.1	1.06	1.07	0.95	1.01	1
p53 (Phospho-Ser392)	0.91	0.95	0.92	0.92	0.94	1.09	1.06	1.01	1.05	0.95	1.07	0.98
p53 (Phospho-Ser6)	1.02	1	1.03	0.92	1.06	1	0.89	0.94	0.99	1.01	0.96	0.91
P70S6K (Ab-229)	0.9	0.94	0.89	1.09	0.99	0.99	1.07	1.06	0.89	0.88	0.88	1.09
P70S6K (Ab-371)	0.9	0.95	0.91	0.98	0.93	0.9	0.9	0.94	0.9	0.94	1.07	1.01
p70S6K (Ab-411)	1.09	0.91	0.9	0.9	0.91	0.9	0.95	0.88	1.03	0.92	0.89	1

P70S6K (Ab-418)	1	0.93	1.11	1.08	0.98	0.91	0.87	0.92	1.07	0.88	0.95	1.1
p70S6k (Ab-421)	0.96	1	1	1	1.04	1.07	1.02	0.89	1.02	1.11	1.06	1.06
p70S6K (Ab-424)	0.95	1.11	0.92	1.09	0.91	0.97	0.94	0.94	1.11	0.97	1.11	1.1
p70S6K (Ab-427)	0.95	0.97	0.9	1.05	1.02	0.94	1.09	0.88	0.98	0.91	0.9	1.06
p70S6K (Phospho-Ser41	0.95	1.11	0.96	1.03	1.12	1.09	0.9	1.03	0.87	1.04	0.98	0.89
p70S6K (Phospho-Ser42	1.05	0.87	0.89	1.09	0.91	0.87	0.92	1.05	1.1	0.99	1.11	0.88
p70S6K (Phospho-Thr22	1.05	0.96	0.96	0.9	1.02	0.94	1.02	0.89	0.9	1.04	1.02	0.92
p70S6K (Phospho-Thr38	1.05	0.97	1.11	1.02	0.92	0.93	1.03	0.97	1	0.98	0.87	1.07
p70S6k (Phospho-Thr42	0.93	0.98	0.93	0.89	0.91	0.98	0.95	1.1	1.01	1.05	0.96	1.12
p70S6K(Phospho-Ser371	0.89	1.04	0.89	0.98	0.98	0.9	1.01	1.03	0.91	1.08	1.03	1.11
p70S6K(Phospho-Ser418	0.88	1.07	1.07	1.1	1	1.01	1.1	1.06	0.96	0.89	1.02	0.94
p70S6K-beta (Ab-423)	1.03	0.87	1.1	0.91	0.94	1.05	0.98	1.1	1.06	0.95	1	0.94
p70S6K-beta (Phospho-Ser423)	1.12	0.91	0.9	1.01	1.12	0.99	0.94	1	1.03	0.92	1.06	0.92
P90RSK (Ab-359/363)	1.05	1.06	0.87	1.04	1.09	0.98	0.98	1.1	0.95	0.93	0.97	1.02
P90RSK (AB-380)	0.92	1.08	1.12	1.05	1.01	1.06	1.06	1.05	0.92	0.91	1.11	0.94
P90RSK (Ab-573)	0.92	0.87	0.93	0.96	1.07	1.11	0.96	1.01	1.06	0.99	0.9	0.99
P90RSK (Phospho-Ser38	1.07	0.94	0.93	0.95	0.98	0.94	1.11	0.9	1.08	1.06	0.98	0.95
P90RSK (Phospho-Thr35	0.92	0.92	1.09	1.06	1.1	0.97	1.07	1.12	1.01	0.93	1.09	1
P90RSK (Phospho-Thr57	1.08	1.1	0.96	0.92	1	0.89	0.97	1.11	1.02	1.08	0.89	1.06
PARP (inter)	0.94	1.02	1.11	0.94	1.08	0.93	1.02	1.04	1.04	0.94	1.11	1.1
PI3-kinase p85-subunit alpha/gamma (Ab-467/199)	1.07	1.11	0.96	1.08	0.96	0.89	1.11	0.95	1.12	1.11	1.06	1.04
PI3-kinase p85-subunit a	0.87	1.07	1.06	0.98	1.12	0.98	0.94	0.89	0.92	0.9	1.01	1.07
PKA CAT (Ab-197)	0.92	1.11	1	0.92	1.09	1.06	1.1	1.1	0.99	0.88	0.87	1.08
PKA CAT (Phospho-Thr1	1.07	1	1.06	0.92	1.01	1.03	1.03	0.96	1.09	1.04	1.05	0.89
PKC pan activation site	0.92	1.07	0.9	1.05	0.91	1.09	1.11	0.96	0.97	1.08	1.08	1.02
PKC pan activation site	0.91	0.9	1.03	0.96	0.91	1.09	0.93	1.01	1	0.93	1	0.9
PTEN (Ab-370)	0.92	0.99	1.09	1.03	1.09	1.08	0.87	0.91	0.88	1.06	1.04	0.99
PTEN (Ab-380)	0.98	0.91	0.91	1.05	0.97	0.89	1.06	1.01	0.94	0.89	1.12	1.06
PTEN (Phospho-Ser370)	0.88	1.03	1	1.06	0.97	0.96	1	0.95	1.12	0.99	0.92	0.93
PTEN (Phospho-Ser380)	1.12	1	1.11	1.09	1	1.11	0.91	0.96	1.11	1.07	0.92	0.95
PTEN (Phospho-Ser380/Thr382/Thr383	0.92	1.01	0.94	1.03	1.11	0.96	1.04	0.9	0.96	0.89	0.88	1.01
PTEN(Ab-	0.97	1.02	1.07	0.9	0.97	0.88	1.06	1.07	0.97	1.02	0.88	1.02
SAPK/JNK (Ab-183)	0.95	0.94	0.88	0.96	0.98	1.12	1.05	1	0.91	1.03	0.88	1.09
SAPK/JNK (Ab-185)	1.06	0.95	0.96	1.04	1.12	0.89	1.04	0.93	1.05	1.1	0.95	0.89
SAPK/JNK (Phospho-Thr	0.92	1.07	1.04	1.04	0.98	1.05	0.88	0.98	0.87	1.04	1	1.11
SAPK/JNK (Phospho-Tyr	0.98	1.08	1.08	0.99	0.96	0.98	1	1.07	1.04	1.08	0.88	1.08
Survivin (Ab-117)	1.01	1.01	1.05	0.88	1.07	0.88	1.01	1.04	0.97	0.91	1.12	0.89
Survivin (Phospho-Thr117)	1.06	0.89	0.99	1.1	1.11	0.95	1.12	1.1	0.97	1.06	1.09	1.09
TAK1 (Ab-184)	1.1	1.12	0.88	1.12	1.02	0.97	1.02	1.04	1.05	1.07	0.96	0.97
TAK1 (Phospho-	0.94	0.88	1.03	1.07	1.07	1.1	0.93	0.94	1.06	0.92	1.05	0.89
TNF Receptor II (C-	0.88	0.99	0.98	1.11	0.87	0.9	0.96	1.02	0.94	0.99	1	0.88
TNF Receptor-1 (inter)	1.04	1.02	0.98	0.91	0.93	1.05	0.94	0.97	0.98	0.87	1.05	1.02
TRADD (inter)	1.06	1.05	0.87	0.96	1	0.99	1.04	0.95	1	0.94	0.95	0.95
XIAP (Ab-87)	0.9	0.89	1.06	1.01	0.97	0.87	0.95	1.1	1.1	1.02	1.06	0.94
XIAP (Phospho-Ser87)	0.95	0.95	1.09	1.08	0.94	1.05	0.87	0.91	1.08	1.09	1.03	0.95

Supplementary Table 3

entry	status	protein_names	gene_names	length	motif	motif_start	motif_type
Q9EPV5	reviewed	Apoptotic prote	Apaf1	1249	KVKSQ	42	phos. act.
Q9EPV5	reviewed	Apoptotic prote	Apaf1	1249	KDRLR	224	acetyl. act.
Q9EPV5	reviewed	Apoptotic prote	Apaf1	1249	QKDVK	390	canonical
Q9EPV5	reviewed	Apoptotic prote	Apaf1	1249	KDVKV	391	acetyl. act.
Q9EPV5	reviewed	Apoptotic prote	Apaf1	1249	DKKVK	678	acetyl. act.
Q9EPV5	reviewed	Apoptotic prote	Apaf1	1249	QTIRV	904	phos. act.
Q9EPV5	reviewed	Apoptotic prote	Apaf1	1249	KDFRL	1048	acetyl. act.
